# Supplementary material for: Do health professionals know about overdiagnosis in screening, and how are they dealing with it? A mixed-methods systematic scoping review
Source: PLoS One. 2025 Feb 3;20(2):e0315247. doi: 10.1371/journal.pone.0315247 (PMC11790174; doi:10.1371/journal.pone.0315247)
Supplement: S1 Table — Eligibility of publications. (DOCX) [file pone.0315247.s001.docx]

| **Author** | **Title** | **Reason for exclusion (if excluded)** | **Assessing perspective on benefits and harms?** |
| --- | --- | --- | --- |
| Akerman et al. | Prostate cancer screening among family physicians in Ontario: An update on attitudes and current practice | Included | yes |
| Allard et al. | Prostate cancer screening: Attitudes and practices of family physicians in Ontario | Not about Odx | yes |
| Alqudah et al. | Early Detection of Prostate Cancer: Self-Reported Knowledge and Attitude of Physicians in Jordan | Not about Odx | no |
| Ambagtsheer et al. | General practitioners' perceptions, attitudes and experiences of frailty and frailty screening | Not about screening | no |
| Amélie et al. | What do women and healthcare professionals expect of decision aids for breast cancer screening? A qualitative study in France | Not about Odx | no |
| Aminsharifi et al | Primary care perspective and implementation of a multidisciplinary, institutional prostate cancer screening algorithm embedded in the electronic health record | Not about Odx | no |
| Araujo et al. | Prostate cancer screening: Beliefs and practices of the Brazilian physicians with different specialties | Not about Odx | no |
| Aziz et al. | Health care providers' perceptions and practices of screening for domestic violence in Upper Egypt | Not about screening | no |
| Bajaj et al. | Screening for suicidal thoughts in primary care: The views of patients and general practitioners | Not about Odx | yes |
| Balekouzou et al. | Assessment of breast cancer knowledge among health workers in Bangui, central African Republic: A cross-sectional study | Not about screening | n/a |
| Bhuyan | Patient-Provider Communication About Prostate Cancer Screening and Treatment: New Evidence From the Health Information National Trends Survey | Not HCPs | n/a |
| Braunschneider et al. | GPs' views on the use of depression screening and GP-targeted feedback: a qualitative study | Not about Odx | yes |
| Brunner et al. | Provider decision-making for prostate cancer screening: A qualitative study | Not retrieved | n/a |
| Burrows et al. | Prostate cancer screening practices amongst physicians in the North Simcoe Muskoka Local Health Integration Network | Not about Odx | no |
| Campbell et al. | Attitudes of healthcare professionals and parents regarding genetic testing for violent traits in childhood | Not about Odx | no |
| Chan et al. | Brief report: Physicians and their personal prostate cancer-screening practices with prostate-specific antigen | Duplicate - same data | n/a |
| Chan et al. | Physician perspectives on the importance of facts men ought to know about prostate-specific antigen testing | Included | yes |
| Chong et al. | Knowledge and practice of breast cancer screening amongst public health nurses in Singapore | Not about Odx | no |
| Clavarino et al. | The view from two sides: a qualitative study of community and medical perspectives on screening for colorectal cancer using FOBT | Not about Odx | no |
| Clements et al. | The PSA testing dilemma: GPs' reports of consultations with asymptomatic men: a qualitative study | Included | no |
| Cmelzer et al. | We Just Never Have Enough Time Clinician Views of Lung Cancer Screening Processes and Implementation" | Not about Odx | no |
| Copp et al. | Clinicians' perspectives on diagnosing polycystic ovary syndrome in Australia: a qualitative study | Not about screening | n/a |
| Dai et al. | Physician Characteristics and Decisions Regarding Cancer Screening: A Systematic Review | SR | n/a |
| Dawson et al. | General practitioners' perceptions of population based bowel screening and their influence on practice: a qualitative study | Not about Odx | no |
| Din et al. | Attitudes toward newborn screening for cytomegalovirus infection | Not HCPs | n/a |
| Dois et al. | [Experts' views on the communication of risks and benefits of mammography to detect breast cancer] | Included | no |
| Elstad et al. | Clinicians' perceptions of the benefits and harms of prostate and colorectal cancer screening | Included | yes |
| Enns et al. | Discontinuing cancer screening for older adults: A comparison of clinician decision-making across breast, prostate, and colon cancer screenings | Not about Odx | yes |
| Enns et al. | Discontinuing Cancer Screening for Older Adults: a Comparison of Clinician Decision-Making for Breast, Colorectal, and Prostate Cancer Screenings | Not about Odx | yes |
| Ersek et al. | Knowledge of, attitudes toward, and use of low-dose computed tomography for lung cancer screening among family physicians | Not about Odx | yes |
| Farrell et al. | How underlying patient beliefs can affect physician-patient communication about prostate-specific antigen testing | Not HCPs | n/a |
| Firzara et al. | Knowledge and practice of prostate cancer screening among general practitioners in Malaysia: a cross-sectional study | Not about Odx | no |
| Gimenez et al. | Representation of overdiagnosis in breast cancer screening among general practitioners: a qualitative study by focus groups | Included | yes |
| Goldenberg et al. | Prostate-specific antigen testing for prostate cancer screening: A national survey of Canadian primary care physicians' opinions and practices | Included | yes |
| Gunn et al. | Using Mixed Methods With Multiple Stakeholders to Inform Development of a Breast Cancer Screening Decision Aid for Women With Limited Health Literacy | Included | yes |
| Han et al. | Decision making in prostate-specific antigen screening - National Health Interview Survey, 2000 | Not HCPs | n/a |
| Hayat Roshanai et al. | Factors influencing primary care physicians' decision to order prostate-specific antigen (PSA) test for men without prostate cancer | Not about Odx | no |
| Henderson et al. | Opinions, practice patterns, and perceived barriers to lung cancer screening among attending and resident primary care physicians | Not about Odx | yes |
| Henderson et al. | Opinions and Practices of Lung Cancer Screening by Physician Specialty | Not about Odx | yes |
| Hersch et al. | Views of healthcare professionals on issues around ductal carcinoma in situ detected through an expanded mammography screening program | Not about screening | n/a |
| Hoffman et al. | Attitudes and Beliefs of Primary Care Providers in New Mexico About Lung Cancer Screening Using Low-Dose Computed Tomography | Not about Odx | yes |
| Hoffman et al. | Decision-making processes for breast, colorectal, and prostate cancer screening: The DECISIONS survey | Not HCPs | n/a |
| Hoffman et al. | Prostate cancer screening decisions: results from the National Survey of Medical Decisions (DECISIONS study) | Not HCPs | n/a |
| Illes et al. | Attitudes towards predictive genetic testing for Alzheimer's disease | Not about Odx | no |
| James et al. | Nephrologists' perspectives on cancer screening in patients with chronic kidney disease: An interview study | Not about Odx | yes |
| Kappen et al. | General practitioners' approaches to prostate-specific antigen testing in the north-east of the Netherlands | Included | yes |
| Kappen et al. | Early detection of prostate cancer using prostate-specific antigen testing: an empirical evaluation among general practitioners and urologists | Included | yes |
| Kappen et al. | Attitudes Toward and Use of Prostate-Specific Antigen Testing Among Urologists and General Practitioners in Germany: A Survey | Included | yes |
| Kerr et al. | Dermatopathologist Perceptions of Overdiagnosis of Melanocytic Skin Lesions and Association With Diagnostic Behaviors | Not about screening | n/a |
| Khairy et al. | An Analysis of Lung Cancer Screening Beliefs and Practice Patterns for Community Providers Compared to Academic Providers | Not about Odx | yes |
| Laza-Vásquez et al. | Views of health professionals on risk-based breast cancer screening and its implementation in the Spanish National Health System: A qualitative discussion group study | Not about Odx | no |
| Lee et al. | Mismatch between health-care professionals' and patients' views on a diabetes patient decision aid: a qualitative study | Not about screening | n/a |
| Lewis et al. | Low-Dose CT Lung Cancer Screening Practices and Attitudes among Primary Care Providers at an Academic Medical Center | Not about Odx | yes |
| Linder et al. | Physician behaviors to promote informed decisions for prostate cancer screening: a National Research Network study | Not about screening | n/a |
| Little et al. | PSA testing in general practice | Not about Odx | no |
| Lowenstein et al. | Real-world lung cancer screening decision-making: Barriers and facilitators | Not about Odx | yes |
| Mainous et al. | Academic family physicians' perception of genetic testing and integration into practice: a CERA study | Not about Odx | no |
| Malli | [Early detection of prostate cancer by PSA testing: the results of a qualitative study on barriers caused by physicians in Austria implementing informed decision making] | Included | yes |
| Marshall et al. | Attitudes of Australian Specialists to Lung Cancer Screening with Low Dose Computed Tomography | Not about Odx | no |
| Martinez et al. | Are Providers Prepared to Engage Younger Women in Shared Decision-Making for Mammography? | Included | yes |
| Maschke et al. | Discussions of Potential Mammography Benefits and Harms among Patients with Limited Health Literacy and Providers: Oh, There are Harms?"" | Not about Odx | no |
| McCormack et al. | Behind Closed Doors: What Happens when Patients and Providers Talk about Prostate-Specific Antigen Screening?: Survey of the Effects of a Community-Based Intervention | Not HCPs | n/a |
| McKinn et al. | Clinician views and experiences of non-invasive prenatal genetic screening tests in Australia | Not about Odx | yes |
| Meloncelli et al. | Clinicians' perspectives on gestational diabetes screening during the global COVID-19 pandemic in Australia | Not about Odx | yes |
| Moss et al. | Prostate-specific antigen testing rates and referral patterns from general practice data in England | Not HCPs | n/a |
| Murphy et al. | The 'general check-up' in the asymptomatic adult—a study of GPs in the North West of Ireland | Not about Odx | no |
| Nguyen et al. | Quebec breast cancer screening program: a study of the perceptions of physicians in Laval, Que | Not about Odx | no |
| O'Brien et al. | Primary care providers' views on a future lung cancer screening program | Not about Odx | no |
| Ontario health technology assessment series | Perspectives of Pregnant People and Clinicians on Noninvasive Prenatal Testing: A Systematic Review and Qualitative Meta-synthesis | SR | n/a |
| Parker et al. | Framing overdiagnosis in breast screening: a qualitative study with Australian experts | Included | yes |
| Parker et al. | The role of communication in breast cancer screening: a qualitative study with Australian experts | Included | yes |
| Parker et al. | Values in breast cancer screening: an empirical study with Australian experts | Included | yes |
| Persaud et al. | Prostate-specific antigen-based screening in Afro-Caribbean men: a survey of members of the Caribbean Urological Association | Not about Odx | no |
| Petrova et al. | Strengths and Gaps in Physicians' Risk Communication: A Scenario Study of the Influence of Numeracy on Cancer Screening Communication | Included | no |
| Petruccelli et al. | A Taxonomy of Reported Harms in Pediatric Autism Spectrum Disorder Screening: Provider and Parent Perspectives | Not about Odx | yes |
| Phillips et al. | A review of studies examining stated preferences for cancer screening | SR | n/a |
| Pickles et al. | Doctors' approaches to PSA testing and overdiagnosis in primary healthcare: a qualitative study | Included | yes |
| Pickles et al. | Doctors' perspectives on PSA testing illuminate established differences in prostate cancer screening rates between Australia and the UK: a qualitative study | Not about Odx | no |
| Ramsay et al. | Should health professionals screen women for domestic violence? Systematic review | SR | n/a |
| Rariden et al. | Screening for Adverse Childhood Experiences: Literature Review and Practice Implications | SR | n/a |
| Raz et al. | Perceptions and Utilization of Lung Cancer Screening Among Primary Care Physicians | Not about Odx | no |
| Roland et al. | Primary care provider practices and beliefs related to cervical cancer screening with the HPV test in Federally Qualified Health Centers | Not about Odx | no |
| Samal et al. | The primary care perspective on routine urine dipstick screening to identify patients with albuminuria | Not a research article | n/a |
| Schlöszler et al. | What attitudes do medical students have towards cancer-screening? [poster] | Not HCPs | n/a |
| Schoenborn et al. | Clinician Perspectives on Overscreening for Cancer in Older Adults With Limited Life Expectancy | Not about Odx | no |
| Schonberg et al. | Primary Care Providers' Perceptions of the Acceptability, Appropriateness, and Feasibility of a Mammography Decision Aid for Women Aged 75 and Older | Included | no |
| Shimada et al. | Knowledge of the potential benefits and harms of breast cancer screening: A survey of participants and nurses | Included | yes |
| Shin et al. | A national survey of lung cancer specialists' views on low-dose CT screening for lung cancer in Korea | Not about Odx | yes |
| Siedlikowski et al. | Physician Perspectives on Mammography Screening for Average-Risk Women: Like a Double-Edged Sword"" | Indirect observation | n/a |
| Siedlikowski et al. | Scrutinizing screening: a critical interpretive review of primary care provider perspectives on mammography decision-making with average-risk women | SR | n/a |
| Sifri et al. | Primary Care Providers' Attitudes and Practices Regarding Cancer Screening in Older Adults | Not about Odx | yes |
| Sijben et al. | DUTCH, UK AND US PROFESSIONALS’ PERCEPTIONS OF SCREENING FOR BARRETT'S ESOPHAGUS AND ESOPHAGEAL ADENOCARCINOMA: A CONCEPT MAPPING STUDY | Not retrieved | n/a |
| Smith et al. | General practitioners' views and experiences of communicating with older people about cancer screening: a qualitative study | Included | no |
| Smith et al. | Physicians' attitudes and behaviour toward screening mammography in women 40 to 49 years of age | Not about Odx | yes |
| Sutton et al. | GPs views and understanding of PSA testing, screening and early detection; survey | Not about Odx | yes |
| Tasian et al. | Prostate specific antigen screening for prostate cancer: knowledge of, attitudes towards, and utilization among primary care physicians | Not about Odx | no |
| Taylor et al. | What are the views of three key stakeholder groups on extending the breast screening interval for low-risk women? A secondary qualitative analysis | Not about Odx | no |
| Thurtle et al. | General practitioner perception of prostate-specific antigen testing has improved, but more awareness of prostate cancer risk in younger patients is still awaited | Not about Odx | no |
| Tisnado et al. | Developing and testing a decision aid for use by providers in making recommendations: about mammography screening in older women | Not about Odx | no |
| Toledo-Chávarri et al. | A qualitative study on a decision aid for breast cancer screening: Views from women and health professionals | Included | no |
| Tudiver et al. | What influences family physicians' cancer screening decisions when practice guidelines are unclear or conflicting? | Not about Odx | no |
| Van Rij et al. | General practitioners attitudes towards PSA screening in asymptomatic men | Not about Odx | no |
| Volk et al. | Primary care physicians' use of an informed decision-making process for prostate cancer screening | Not about screening | no |
| Voss et al. | Prostate cancer screening practices and beliefs | Not about Odx | no |
| Walters et al. | Health care professionals' preferences for extending mammographic breast screening to the over 70s | Included | yes |
| Wang et al. | Understanding professional stakeholders’ active resistance to guideline implementation: The case of Canadian breast screening guidelines | Not a research article | n/a |
| Wegwarth et al. | There is nothing to worry about": gynecologists' counseling on mammography" | Indirect observation | n/a |
| Wegwarth et al. | Less is more: Overdiagnosis and overtreatment: evaluation of what physicians tell their patients about screening harms | Indirect observation | n/a |
| Wegwarth et al. | US gynecologists' estimates and beliefs regarding ovarian cancer screening's effectiveness 5 years after release of the PLCO evidence | Not about Odx | yes |
| Whelehan et al. | Client and practitioner perspectives on the screening mammography experience | Not about screening | n/a |
| Williams et al. | Screening and Brief Interventions for Alcohol and Other Drug Use Among Pregnant Women Attending Midwife Obstetric Units in Cape Town, South Africa: A Qualitative Study of the Views of Health Care Professionals | Not about Odx | Benefits, not harms |
| Woodrow et al. | Bowel cancer screening in England: a qualitative study of GPs' attitudes and information needs | Not about Odx | yes |
| Woof et al. | Introducing a low-risk breast screening pathway into the NHS Breast Screening Programme: Views from healthcare professionals who are delivering risk-stratified screening | Not about Odx | no |
| Yasmeen et al. | Screening mammography beliefs and recommendations: a web-based survey of primary care physicians | Not about Odx | no |
| Zapka et al. | Physicians' colorectal cancer screening discussion and recommendation patterns | Not about Odx | no |
