# Supplementary material for: Do health professionals know about overdiagnosis in screening, and how are they dealing with it? A mixed-methods systematic scoping review
Source: PLoS One. 2025 Feb 3;20(2):e0315247. doi: 10.1371/journal.pone.0315247 (PMC11790174; doi:10.1371/journal.pone.0315247)

# Appendix

Table 1: relevant abstracts from the Preventing Overdiagnosis conferences

| **Year** | **Author** | **Titel** | **In database search?** | **Author response** |
| --- | --- | --- | --- | --- |
| 2013 | Maihan VU | Abstract #63 - HOW DO PRIMARY CARE PHYSICIANS WEIGH RECOMMENDATIONS TO STOP PSA SCREENING AND PATIENTS’ REQUESTS TO BE SCREENED? | yes | n/a |
| 2013 | Kirsten Pickles | Abstract #64 -DRIVERS OF OVERDIAGNOSIS IN PROSTATE CANCER SCREENING: AN AUSTRALIAN GP PERSPECTIVE | yes | n/a |
| 2014 | Kirsten Pickles | #67 - Approaches to PSA testing in Australian general practice: a new empirical analysis of overdiagnosis and the personal burden experienced by doctors | yes | n/a |
| 2014 | Emily DeVoto | Small Facebook survey | no | yes: data not published |
| 2015 | Johanna Caro Mendivelso | 0136 - Perceptions and attitudes of primary care physicians toward overdiagnosis and unnecessary care. Anna Kotzeva, Johanna Caro, Nuria Prat, Cristina Adroher, Cari Almazan | no | no |
| 2015 | Ozge KARANFIL | 0167 - Risk Perception, Preferences and Behaviors in Prostate Cancer Screening: A System without Negative Feedback Ozge Karanfil, John D. Sterman | no | no |
| 2016 | Kathrin Schlößler | A German Decision Aid to use within pre-screening discussions-A mixed methods study on patient and physician perspectives | no | no |
| 2017 | Thomas Kuhlein | How do German GPs think about overdiagnosis - a questionnaire study. Susann Schaffer, Maximilian Pausch, Thomas Kühlein, Angela Schedlbauer | yes, but not about overdiagnosis | no publication about overdiagnosis |
| 2017 | Guylène Thériault | Poster: Understanding & Taking Action | no | yes - no formal report |
| 2022 | Smith | 56 General practitioner’s attitudes and behaviours regarding cancer screening in older adults: A qualitative interview study | yes | n/a |

Chcecklist 1 quality appraisal Delphi study by Gunn et al.


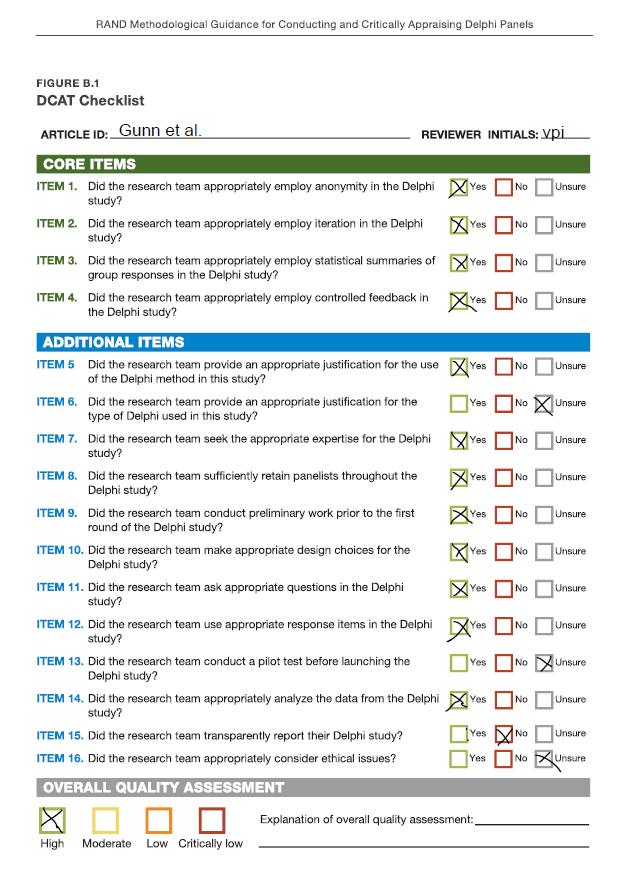

Supplement: S1 Appendix — Selection QA. (DOCX) [file pone.0315247.s002.docx]
