# Supplementary material for: Do health professionals know about overdiagnosis in screening, and how are they dealing with it? A mixed-methods systematic scoping review
Source: PLoS One. 2025 Feb 3;20(2):e0315247. doi: 10.1371/journal.pone.0315247 (PMC11790174; doi:10.1371/journal.pone.0315247)
Supplement: S2 Table — QN results. (DOCX) [file pone.0315247.s003.docx]

***QUANTITATIVE RESULTS***

Table 1: quantitative studies that report awareness of overdiagnosis among health professionals

| Reference | Country | Screening topic | Participants | Number of participants | % awareness | *Survey question* |
| --- | --- | --- | --- | --- | --- | --- |
| Akerman et al., 2018 [73] | Canada | Prostate cancer | General Practitioners | 1880 | 73% | *Screening with PSA leads to overdiagnosis and overtreatment of prostate cancer: agree* |
| Elstad et al. , 2015 [75] | USA | Prostate & colorectal cancer | Primary care clinicians | 126 | 28% (PSA)  8% (colonoscopy) | *How many clinicians mention overdiagnosis as a possible harm of screening with PSA/colonoscopy?* |
| Martinez et al. , 2018 [81] | USA | Breast cancer | Primary care clinicians | 220 | 92% | *If a woman aged 40–49 with average risk undergoes screening mammography, what is the chance that she will be treated for a breast cancer that would never have caused problems if left untreated? (% of PCP's that indicates 1 or more women)* |
| Shimada et al., 2017 [86] | Japan | Breast cancer | Nurses working in a breast screening clinic | 1710 | 57% | *Do you know that there are some slow-growing cancers that do not immediately cause death? % of nurses with correct answer?* |
| Walters et al., 2010 [72] | UK | Breast cancer | Breast cancer or geriatric experts | 139 | 59% | *The Breast Screening program may cause harm by overdiagnosing cancers/DCIS - Agree* |

Table 2: quantitative studies that report health professionals’ ideas about informing people about overdiagnosis before screening
