# Supplementary material for: Do health professionals know about overdiagnosis in screening, and how are they dealing with it? A mixed-methods systematic scoping review
Source: PLoS One. 2025 Feb 3;20(2):e0315247. doi: 10.1371/journal.pone.0315247 (PMC11790174; doi:10.1371/journal.pone.0315247)
Supplement: S3 Table — Data extraction by publication. (DOCX) [file pone.0315247.s004.docx]

**DATA-EXTRACTION PER PUBLICATION**

**Missing data:**

- Quantitative studies: missing data are only seldomly reported. We copied the reported information in Table 1, column “Responders for this question”.
- Missing data: not applicable for qualitative research

**Table 1 – Data extraction quantitative studies**

Abbreviations: IHC: Individual Healthcare, PH: Public Health, GP: General Practitioner, PC: Primary Care, NR= not reported

| **Reference** | **Eligibility & data extraction** | **Setting & Design** | **Research question (study)** | **Extracted question (+ response categories)** | **Health professional** | **Invited** | **Responders (%)** | **Responders for this question (N)** | N | % |
| --- | --- | --- | --- | --- | --- | --- | --- | --- | --- | --- |
| **Akerman et al., 2018 [1]** | - Eligible for inclusion: YES - Data extracted by: Veerle Piessens - Date of data-extraction: april 2022 | - Canada - IHC - GPs - Prostate cancer - Questionnaire | Our present study seeks to re-evaluate prostate cancer screening practices among PCPs in Ontario since the publication of new Canadian and American task force recommendations. 1) beliefs and attitudes with respect to the utility of prostate cancer screening; 2) current screening practices for prostate cancer; and 3) awareness and knowledge of North American recommendations/guidelines for prostate cancer screening. | *Screening with PSA leads to overdiagnosis and overtreatment of prostate cancer:* ***% of participants that agreed?*** | *GPs* | 11.657 | 1.880 (16,1%) | *NR* | *NR* | 72,6% |
| **Chan et al., 2003 [2]** | - Eligible for inclusion: YES - Data extracted by: Veerle Piessens - Date of data-extraction: april 2022 | - USA - IHC - Physicians - Prostate cancer - Questionnaire | To determine physicians’ rating of the importance of key facts men ought to know about prostate-specific antigen (PSA) screening and whether there are differences by specialty (urologists, internists, family physicians). | *Prostate cancer may grow slowly and not cause symptoms. That is why it may not kill older men. They may outlive this cancer and die from something else.* ***How many non-urologists rated this fact important for asymptomatic men to know before deciding on getting a PSA test?*** | *Non-urologists* | 522 | 299  (57,3%) | *NR* | *NR* | 90,0% |
|  |  |  |  | *"Prostate cancer may grow slowly and not cause symptoms. That is why it may not kill older men. They may outlive this cancer and die from something else.* ***How many urologists rated this fact important for asymptomatic men to know before deciding on getting a PSA test?*** | *Urologists* | 247 | 151  (61,1%) | *NR* | *NR* | 79% |
| **Elstad et al. , 2015 [3]** | - Eligible for inclusion: YES - Data extracted by: Veerle Piessens - Date of data-extraction: april 2022 | - USA - IHC - Primary care clinicians - Prostate & Colorectal cancer - Questionnaire | The study had 2 distinct but complementary aims. First, we sought to describe clinicians’ perceptions of the specific benefits and harms of our 2 chosen screening services, including the number and perceived magnitude of benefits and harms they could call to mind. Second, we sought to understand how clinicians arrive at their perceptions of the likelihood of life lengthened or harm from screening. | *How many clinicians mention overdiagnosis as a possible harm of screening with PSA?* | *Primary care clinicians* | 155 | 126  (81,3%) | *NR* | *NR* | 28,0% |
|  |  |  |  | *If clinicians mention overdiagnosis as a possible harm of screening with PSA,* ***which magnitude do they give to the risk? (1= almost none - 4= very high)*** |  |  |  | *NR* | *NR* | 3,48 |
|  |  |  |  | *How many clinicians mention overdiagnosis as a possible harm of screening with colonoscopy?* |  |  |  | *NR* | *NR* | 8,0% |
|  |  |  |  | *If clinicians mention overdiagnosis as a possible harm of screening with colonoscopy,* ***which magnitude do they give to the risk? (1= almost none - 4= very high)*** |  |  |  | *NR* | *NR* | 2,67 |
| **Goldenberg et al., 2017 [4]** | - Eligible for inclusion: YES - Data extracted by: Veerle Piessens - Date of data-extraction: april 2022 | - Canada - IHC - Primary Care physicians - Prostate cancer - Questionnaire | We created a survey instrument to survey a national sample of Canadian PCPs to understand their knowledge of and agreement with the CTFPHC guidelines, their current screening practices, and their use of shared decision-making around PSA testing. | *"When discussing the PSA test and prostate cancer screening with patients, which potential harms of screening do you routinely mention?"* ***% of PCPs that selected 'overdiagnosis'*** | *Primary care physicians* | 19633 | 1190  (6,1%) | NR | NR | 77,30% |
| **Gunn et al. , 2021 [5]** | - Eligible for inclusion: YES - Data extracted by: Veerle Piessens - Date of data-extraction: april 2022 | - USA - PH - Experts in breast screening, decision making or health literacy - Breast cancer - Delphi | This analysis focuses specifically on triangulating perspectives to understand areas of divergence and convergence across stakeholder groups (experts, primary care providers [PCPs], and patients with LHL) that require synthesis in developing DAs for breast cancer screening among limited health literacy populations who have been historically underrepresented in the design process. | *How important is it that a breast cancer screening decision aid for women with limited health literacy describes overdiagnosis as a possible screening harm?* ***% that answers very important*** | *Experts in breast screening, decision making or health literacy* | 15 | 8  (53,3%) | *NR* | *7* | 88% |
|  |  |  |  | *How important is it that a breast cancer screening decision aid for women with limited health literacy describes the overdiagnosis rate?* ***% that answers very important*** |  |  |  | NR | 6 | 75% |
| **Kappen et al. , 2019 [6]** | - Eligible for inclusion: YES - Data extracted by: Veerle Piessens - Date of data-extraction: april 2022 | - Germany - IHC - GPs & urologists - Prostate cancer - Questionnaire | ...to investigate potential differences in daily routine practice regarding PSA testing between German GPs/internists and urologists | *Would you discuss potential* ***overdiagnosis*** *before performing a PSA-test?* ***% of GPs responding 'often' or 'always'?*** | *GPs* | 172 | 41  (23,8%) | *NR* | *33* | 80,50% |
|  |  |  |  | *Would you discuss potential* ***overdiagnosis*** *before performing a PSA-test?* ***% of urologists responding 'often' or 'always'?*** | *Urologists* | 128 | 14  (10,9%) | *NR* | *11* | 78,60% |
| **Kappen et al. , 2020 [7]** | - Eligible for inclusion: YES - Data extracted by: Veerle Piessens - Date of data-extraction: april 2022 | - The Netherlands - IHC - GPs - Prostate cancer - Questionnaire | In the present study, we aimed to survey GPs in the Netherlands to assess their approaches, attitudes, and knowledge regarding the use of PSA screening for early prostate cancer detection, focusing on the prostate cancer recommendations set out in the NHG guideline. | *In case I will refer a patient for a PSA test, beforehand, I will discuss with my patient:* ***overdiagnosis*** *as a possible disadvantage of screening****: % of GPs responding 'often' or 'always'?*** | *GPs* | 202 | 88  (43,6%) | *NR* | *64* | 72,7% |
| **Kappen et al. , 2021 [8]** | - Eligible for inclusion: YES - Data extracted by: Veerle Piessens - Date of data-extraction: april 2022 | - Germany - IHC - GPs & urologists - Prostate cancer - Questionnaire | Therefore, to inform implementation of the mammography DA, we aimed to learn PCPs’ perspectives on the DA’s acceptability (palatability), appropriateness (fit), and feasibility (workability). | *Would you discuss potential* ***overdiagnosis*** *before performing a PSA-test?* ***% of GPs responding 'often' or 'always'?*** | *GPs* | 1.579 | 96  (6,1%) | *NR* | *66* | 68,7% |
|  |  |  |  | *Would you discuss potential* ***overdiagnosis*** *before performing a PSA-test?* ***% of urologists responding 'often' or 'always'?*** | *Urologists* | 6.568 | 432  (6,6%) | *NR* | *314* | 72,6% |
| **Martinez et al. , 2018 [9]** | - Eligible for inclusion: YES - Data extracted by: Veerle Piessens - Date of data-extraction: june 2023 | - USA - IHC - PC Clinicians - Breast Cancer - Questionnaire | The objective of this study was to characterize provider readiness to engage younger women in SDM for mammography by evaluating whether providers had adequate knowledge, time, and self-assessed competence to do so. | *If a woman aged 40–49 with average risk undergoes screening mammography, what is the chance that she will be treated for a breast cancer that would never have caused problems if left untreated?* ***(% of PCP's that indicates 1 or more women)*** | *Primary care clinicians* | 612 | 220  (35,9%) | NR | 204 | 92,0% |
| **Petrova et al., 2017 [10]** | - Eligible for inclusion: YES - Data extracted by: Veerle Piessens - Date of data-extraction: april 2022 | - UK - IHC - GPs - Hypothetical cancer - Questionnaire | We investigated whether physicians adapt their risk communication to accommodate the needs of patients with low numeracy, and how physicians’ own numeracy influences their understanding and communication of screening statistics. | *How many FPs would address* ***overdiagnosis*** *in their communication with patients about a fictitious cancer screening.* | *GPs* | 516 | 151  (29,3%) | *NR* | *125* | 82,8% |
| **Schoenberg et al., 2022 [11]** | - Eligible for inclusion: YES - Data extracted by: Veerle Piessens - Date of data-extraction: june 2023 | - USA - IHC - PC Physicians - Breast cancer - Questionnaire | Therefore, to inform implementation of the mammography DA, we aimed to learn PCPs’ perspectives on the DA’s acceptability (palatability), appropriateness (fit), and feasibility (workability). | *How often do you discuss overdiagnosis when talking about mammography screening with women 75+?* ***% that states 'sometimes' or 'frequently/always'*** | *Primary care physicians* | 137 | 80  (58,4%) | NR | 59 | 75% |
| **Shimada et al., 2017 [12]** | - Eligible for inclusion: YES - Data extracted by: Veerle Piessens - Date of data-extraction: april 2022 | - Japan - IHC - Nurses working in a breast screening practice - Breast cancer - Questionnaire | The aim of this study was to determine the knowledge of the general public and of nurses in breast screening practices in Japan, regarding the benefits and risks of breast cancer screening. | *Do you know that there are some slow-growing cancers that do not immediately cause death****? % of nurse with correct answer?*** | *Nurses working in a breast screening practice* | 1.905 | 1.710  (89,8%) | *NR* | *971* | 56,8% |
| **Walters et al., 2010 [13]** | - Eligible for inclusion: YES - Data extracted by: Veerle Piessens - Date of data-extraction: april 2022 | - UK - IHC + PH - Breast cancer or geriatric experts - Mixed methods: questionnaire (+interview) | Are HCPs in favour of extending breast cancer screening beyond the age of 70? If yes, to whom and how should screening be offered? Can HCPs accurately assess which older women may benefit form extended screening? | *The Breast Screening program may cause harm by overdiagnosing cancers/DCIS -* ***% of participants that agreed?*** | *Breast cancer or geriatric experts* | 488 | 139  (28,5%) | *138* | *NR* | 59,0% |
|  |  |  |  | *The Breast Screening program may cause harm by overdiagnosing cancers/DCIS -* ***% of participants that agreed?*** |  |  |  | *138* | *NR* | 25,0% |

**Table 2 - Data-extraction qualitative studies**

Abbreviations: GP = General Practitioner, ODx = overdiagnosis, ORx = overtreatment

| **Reference** | **Eligibility & data extraction** | **Setting & design** | **Research question** | **AWARENESS & KNOWLEDGE** | **SIGNIFICANCE** | **COMMUNICATION** | **SCREENING POLICY** |
| --- | --- | --- | --- | --- | --- | --- | --- |
| **Clements et al., 2007 [14]** | - Eligible for inclusion: YES - Data extracted by: Veerle Piessens - Date of data-extraction: april 2022 | - UK - 2007 - Individual healthcare perspective - GPs - Prostate cancer - Individual interviews | Which discussions do GPs report having with asymptomatic men, prior to PSA-testing? Which factors influence the content of these discussions (according to the GPs)? How well do reported discussions align with the national guidance provided for counseling prior to PSA-testing? | 1. Indirect indication of knowledge (from results & quotes) |  | 1. ODx less frequently addressed compared to false + and false - 2. Not mentioning ODx because of:    1. Indivdual assessment that benefit > ODx risk    2. Belief that it’s the urologists responsibility |  |
| **Dois et al., 2021 [15]** | - Eligible for inclusion: YES - Data extracted by: Veerle Piessens - Date of data-extraction: feb 2023 | - Chili - 2021 - Public health perspective - Breast cancer screening experts - Breast cancer - Focus groups | To describe the opinion of professional experts on what considerations should be taken when providing information to women who are facing the decision of whether or not to undergo mammography | 1. Indirect indication of knowledge (from results & quotes) | / | 1. Some participants believe women should be informed about ODx, however others seem to disagree. | / |
| **Gimenez, 2018 [16]** | - Eligible for inclusion: YES - Data extracted by: Veerle Piessens - Date of data-extraction: april 2022 | - France - 2018 - Individual healthcare perspective - GPs - Breast cancer - Focus groups | To examine the perception of overdiagnosis due to screening among GPs | 1. ODx seems a poorly known concept, confused with false positives 2. Learning about ODx (as a harm of screening, contrasting info compared to current guidelines) causes discomfort | 1. ODx might be reason not to screen (for patients) | 1. Different ideas about informing patients about Odx 2. GPs see themselves as an important, trustworthy source of info for their patients 3. Need for clear material to support communication | 1. Option of individualized screening, after concertation between GP and patient |
| **Malli, 2013 [17]** | - Eligible for inclusion: YES - Data extracted by: Veerle Piessens - Date of data-extraction: pril 2022 | - Austria - 2013 - Individual healthcare perspective - GPs - Prostate cancer - Individual interviews | The aim of this qualitative pilot-study is to explore factors, which provide insight into GPs’ practice of counselling about the PSA test |  |  | None of the participants mention overdiagnosis as an issue to address in SDM. (Explicitely mentioned as a result) |  |
| **Parker et al., 2015 [18]** | - Eligible for inclusion: YES - Data extracted by: Veerle Piessens - Date of data-extraction: pril 2022 | Idem | 1/What are the views of Australian experts about what and how we should communicate with consumers about breast screening? 2/How do experts reason about this topic and how does this explain the positions they take?? | 1. Silent assumption that participants know what Odx is 🡪 No direct questions about awareness or knowledge. 2. Concept of Odx seen as an academic construct, not in line in what (allegedly) matters to patients. Seen as not scientific. | 1. Odx = harm (and citizens should be warned about this risk) 2. Orx = the problem, not Odx as such 3. Odx is a possible harm, but importance (both in quality and quantity) has to be assessed by the individual screening candidate , therefore it is important that citizens are well informed before participating in screening. 4. Odx is a normal (intended) consequence of screening 5. Odx is not a harm (that matters to patients), but a fictitious, academic construct 6. Odx is beneficial | 1. Opposing ideas about informing the public about Odx 2. Pro informing:    1. Because they believe in supporting informed choice an giving full info on Odx is instrumental in supporting informed choice    2. Because there are risks and women have the right to know 3. Against informing (or providing limited information + reassuring that the risk for Odx is low)    1. Because it might scare women away from participation, and as such avail them from having a beneficial intervention    2. Because it is impossible to tell on the individual level whether a screen detected cancer would be overdiagnosed (not actionable information) 4. Different positions towards the importance of informed choice before participating in screening    1. IC = very important    2. IC = unattainable | / |
| **Parker et al., 2015 [19]** | - Eligible for inclusion: YES - Data extracted by: Veerle Piessens - Date of data-extraction: pril 2022 | - Australia - 2015 - Public health perspective - Breast cancer screening experts - Breast cancer - Individual interviews | 1/ How do Australian breast screening experts frame overdiagnosis?  2/ How do those frames present the problems, causal elements, value judgements and solutions relevant to overdiagnosis?” | 1. Silent assumption that participants know what Odx is 🡪 No direct questions about awareness or knowledge. 2. Indirect indication of knowledge (from results & quotes) 3. Complex and hard to grasp phenomenon 4. Different opinions about Odx-rate 5. Concerns about scientific base of knowledge about Odx 6. Discussion of underlying cause of Odx | 1. Odx = harm    1. high number of overdiagnosed cases    2. Burden of diagnosis    3. Burden of treatment 2. Orx = problem (harm), not Odx as such 3. Relevance of Odx = assessed in relation to benefits of screening 4. Odx = minor problem 5. Odx =normal consequence of screening 6. Odx = beneficial | 1. Different ideas about informing the public about Odx 2. Different positions towards making an informed choice to participate in screening 3. Problematic (biased) evidence translation | 1. Risk of Odx should be minimized by avoiding screening 2. Minimize harm by targeted screening of higher risk groups 3. Focus on reducing downstream treatment harms 4. Ensure screening participation, therefore avoid public debate about Odx 5. It’s not for experts to decide 🡪 seek public involvement |
| **Parker et al., 2015 [20]** | - Eligible for inclusion: YES - Data extracted by: Veerle Piessens - Date of data-extraction: pril 2022 | Idem | 1/ What are the values expressed in the talk of Australian experts about breast screening in Australia?  2/What are the implications for policy and practice of experts holding particular values? | 1. Silent assumption that participants know what Odx is 🡪 No direct questions about awareness or knowledge. 2. Concerns and differing opinions about scientific base for screening (including Odx) | 1. Availability bias, related to the professional position of the participants, affected whether or not Odx was conceptualized as a harm. 2. Odx = serious harm, although differing opinions about how high the Odx risk is. 3. Some participants contested the idea that Odx would be a harm. | 1. Opposing ideas about informing the public about Odx    1. Pro informing to support women’s’ autonomy    2. Against informing to avoid scaring women away from participation | 1. Position towards offering screening depends on participants’ conceptualization of benefits and harms and their beliefs about the magnitude of them.    1. Pro screening: delivering benefits is prioritised over avoiding harm, both in relevance as in magnitude(risk of ODx is not considered or estimated as low)    2. Avoiding screening: avoiding harm is prioritised over delivering benefits (ODx is seen as important harm). |
| **Pickles et al., 2015 [21]** | - Eligible for inclusion: YES - Data extracted by: Veerle Piessens - Date of data-extraction: april 2022 | - Australia - 2015 - Individual healthcare perspective - GPs - Prostate cancer - Individual interviews | (1) To explain general practitioners’ (GPs’) approaches to prostate-specific antigen (PSA) testing and overdiagnosis; (2) to explain how GPs reason about their PSA testing routines and (3) to explain how these routines influence GPs’ personal experience as clinicians. | 1. Silent assumption that GPs know what ODx is 🡪 Q about awareness, but no results. 2. Indirect indication of knowledge (from results & quotes) 3. Complex and hard to grasp phenomenon 4. Different positions towards the value and trustworthiness of evidence and research | 1. Some GPs don’t think about ODx 2. ODx = harm 3. ORx = problem (harm), not ODx as such 4. ODx = necessary evil 5. ODx should be seen in relation to benefits of screening | 1. Different communication strategies, from ‘no info’, over tailored messages to full info about ODx. 2. Different ideas about their role in informing about ODx 3. No explicit ideas about informed choice, more focus on shared decision making 4. Communication about ODX perceived as difficult & complex | 1. ODx affects screening policy    1. Avoid screening because of ODx    2. Decision based on patient profile and needs 2. ODx has no effect on screening decisions:    1. Offer screening despite ODx    2. ODx not in their mind |
| **Smith et al., 2022 [22]** |  | - Australia - 2021 - individual healthcare perspective - GPs - Prostate, breast, colorectal, cervical - Indovdual interviews | to investigate GPs’ views and experiences of communication and decision-making about cancer screening (breast, cervical, bowel, and prostate) for older people aged ≥70 years. | 1. Indirect indication of knowledge (from results & quotes) | 1. Seen as a problem because some GPs feel the need to warn their patients | 1. Some GPs mention ODx (indicrectly) when discussing benefits & harms of screening | / |
| **Toledo-Chavarri et al., 2017 [23]** | - Eligible for inclusion: YES - Data extracted by: Veerle Piessens - Date of data-extraction: april 2022 | - Spain - 2017 - Public Health - HCPs involved in breast cancer screening - Breast cancer - Focus groups | … to evaluate a DA that includes the benefits and harms of breast cancer screening and analyse women’s perceptions of the information received and healthcare professionals’ perceptions regarding the convenience of providing it | 1. No direct questions about awareness or knowledge of ODx (not the study objective) . 2. Indirect indication of knowledge (from results & quotes)    1. Correct knowledge    2. Other definition: “looks like ODx , but cancer was entirely removed through diagnostic biopsy” | 1. ODx = harm 2. Burden of diagnosis 3. Burden of treatment 4. ODx =normal consequence of screening 5. ODx = wrongly conceptualised: “seems like ODx , but cancer was entirely removed through diagnostic biopsy” | 1. Two opposing positions towards obligation to inform the public about ODx 2. Pro informing: Moral obligation + Sufficient evidence that shows harm of screening and suggests a narrow balance of benefits and harms 3. Against informing: might deter people for participating in screening + Evidence for benefits of screening is overwhelming + individuals should not question the benefits of screening 4. Different positions towards making an informed choice to participate in screening  - Importance of shared decision making between doctor and woman - Against informed choice:   Evidence for benefits of screening is overwhelming + there is a screening program in place (based on the evidence of the benefits of screening).   1. Evidence translation is difficult: already hard to understand for professionals, must be even harder for the lay public | / |
| **Walters et al., 2010 [13]** | - Eligible for inclusion: YES - Data extracted by: Veerle Piessens - Date of data-extraction: april 2022 | - UK - IHC + PH - Breast cancer or geriatric experts - Mixed methods: (questionnaire) +interview | Are HCPs in favour of extending breastcancerscreening beyond the age of 70? If yes, to whom and how should screening be offered? Can HCPs accurately assess which older women may benefit form extended screening? | Indirect indication of correct knowledge (from results & quotes) | 1. ODx = harm 2. ODx = necessary evil | / | / |

1. Akerman JP, Allard CB, Tajzler C, Kapoor A. Prostate cancer screening among family physicians in Ontario: An update on attitudes and current practice. Can Urol Assoc J. 2018;12(2):E53-e8.

2. Chan ECY, Vernon SW, Haynes MC, O'Donnell FT, Ahn C. Physician perspectives on the importance of facts men ought to know about prostate-specific antigen testing. J Gen Intern Med. 2003;18(5):350-6.

3. Elstad EA, Sutkowi-Hemstreet A, Sheridan SL, Vu M, Harris R, Reyna VF, et al. Clinicians' perceptions of the benefits and harms of prostate and colorectal cancer screening. Medical decision making : an international journal of the Society for Medical Decision Making. 2015;35(4):467-76.

4. Goldenberg MG, Skeldon S, Nayan M, Suppiah Y, Chow L, Fryml E, et al. Prostate-specific antigen testing for prostate cancer screening: A national survey of Canadian primary care physicians' opinions and practices in 2016. Canadian Urological Association Journal. 2017;11(6):S198.

5. Gunn CM, Maschke A, Paasche-Orlow MK, Housten AJ, Kressin NR, Schonberg MA, et al. Using Mixed Methods With Multiple Stakeholders to Inform Development of a Breast Cancer Screening Decision Aid for Women With Limited Health Literacy. MDM Policy and Practice. 2021;6(2).

6. Kappen S, Jürgens V, Freitag MH, Winter A. Early detection of prostate cancer using prostate-specific antigen testing: an empirical evaluation among general practitioners and urologists. Cancer Manag Res. 2019;11:3079-97.

7. Kappen S, Koops L, Jürgens V, Freitag MH, Blanker MH, Timmer A, et al. General practitioners' approaches to prostate-specific antigen testing in the north-east of the Netherlands. BMC Fam Pract. 2020;21(1):270-.

8. Kappen S, Jürgens V, Freitag MH, Winter A. Attitudes Toward and Use of Prostate-Specific Antigen Testing Among Urologists and General Practitioners in Germany: A Survey. Front Oncol. 2021;11:691197.

9. Martinez KA, Deshpande A, Ruff AL, Bolen SD, Teng K, Rothberg MB. Are Providers Prepared to Engage Younger Women in Shared Decision-Making for Mammography? Journal of Womens Health. 2018;27(1):24-31.

10. Petrova D, Kostopoulou O, Delaney BC, Cokely ET, Garcia-Retamero R. Strengths and Gaps in Physicians' Risk Communication: A Scenario Study of the Influence of Numeracy on Cancer Screening Communication. Medical decision making : an international journal of the Society for Medical Decision Making. 2018;38(3):355-65.

11. Schonberg MA, Hamel MB, Davis RB, Karamourtopoulos M, Pinheiro A, Hayes MC, et al. Primary Care Providers' Perceptions of the Acceptability, Appropriateness, and Feasibility of a Mammography Decision Aid for Women Aged 75 and Older. Mdm Policy & Practice. 2022;7(1):1-12.

12. Shimada T, Takahashi M, Tsukisawa K, Shimizu Y, Tanaka M, Saito N, et al. Knowledge of the potential benefits and harms of breast cancer screening: A survey of participants and nurses. Cancer Res. 2016;76(4).

13. Walters SJ, Winslow M, Collins K, Robinson T, Green T, Madan J, et al. Health care professionals' preferences for extending mammographic breast screening to the over 70s. J Geriatr Oncol. 2011;2(1):1-10.

14. Clements A, Watson E, Rai T, Bukach C, Shine B, Austoker J. The PSA testing dilemma: GPs' reports of consultations with asymptomatic men: a qualitative study. BMC Fam Pract. 2007;8:1-7.

15. Dois A, Bravo P, Fernández-González L, Uribe C. [Experts' views on the communication of risks and benefits of mammography to detect breast cancer]. Rev Med Chil. 2021;149(2):196-202.

16. Gimenez L, Janczewski A. Representation of overdiagnosis in breast cancer screening among general practitioners: a qualitative study by focus groups. Exercer-La Revue Francophone De Medecine Generale. 2018(139):18-9.

17. Malli G. [Early detection of prostate cancer by PSA testing: the results of a qualitative study on barriers caused by physicians in Austria implementing informed decision making]. Gesundheitswesen (Bundesverband der Arzte des Offentlichen Gesundheitsdienstes (Germany)). 2013;75(1):22-8.

18. Parker LM, Rychetnik L, Carter SM. The role of communication in breast cancer screening: a qualitative study with Australian experts. BMC Cancer. 2015;15:741.

19. Parker LM, Rychetnik L, Carter S. Framing overdiagnosis in breast screening: a qualitative study with Australian experts. BMC Cancer. 2015;15:606.

20. Parker L, Rychetnik L, Carter S. Values in breast cancer screening: an empirical study with Australian experts. BMJ open. 2015;5(5):e006333.

21. Pickles K, Carter SM, Rychetnik L. Doctors' approaches to PSA testing and overdiagnosis in primary healthcare: a qualitative study. BMJ open. 2015;5(3):e006367.

22. Smith J, Dodd RH, Wallis KA, Naganathan V, Cvejic E, Jansen J, et al. General practitioners' views and experiences of communicating with older people about cancer screening: a qualitative study. Fam Pract. 2022.

23. Toledo-Chávarri A, Rué M, Codern-Bové N, Carles-Lavila M, Perestelo-Pérez L, Pérez-Lacasta MJ, et al. A qualitative study on a decision aid for breast cancer screening: Views from women and health professionals. European Journal of Cancer Care. 2017;26(3).
