# Supplementary material for: Do health professionals know about overdiagnosis in screening, and how are they dealing with it? A mixed-methods systematic scoping review
Source: PLoS One. 2025 Feb 3;20(2):e0315247. doi: 10.1371/journal.pone.0315247 (PMC11790174; doi:10.1371/journal.pone.0315247)
Supplement: S4 Table — Data extraction by review finding. (DOCX) [file pone.0315247.s005.docx]

# DATA-EXTRACTION PER REVIEW FINDING

# Awareness & knowledge

## *None of the qualitative studies investigated what HCPs understood under the term ‘overdiagnosis’*

| **Finding** | **Studies contributing to this finding** | **Quote / Text** |
| --- | --- | --- |
| None of the qualitative studies investigated what HCPs understood under the term ‘overdiagnosis’ | All |  |

## *ODx is poorly understood and confused with false positives*

| **Finding** | **Studies contributing to this finding** | **Quote / Text** |
| --- | --- | --- |
| ODx is poorly understood and confused with false positives | Gimenez[1] | [Text] “Overdiagnosis of breast cancer was a poorly understood and little known concept. It was confused with false positives. It seemed to be better known for prostate cancer” [Gimenez].  [Text] Overdiagnosis is hard to understand for GPs and for the public—and it is contradictory to many people’s existing health beliefs. [Pickles] |

## *When investigators do not prompt the issue of overdiagnosis, only few or even no participants mention it*

| **Finding** | **Studies contributing to this finding** | **Quote / Text** |
| --- | --- | --- |
| When investigators do not prompt the issue of overdiagnosis, only few or even no participants mention it | Malli[2]  Dois[3]  Smith[4] | Quote: "But I think it [overdiagnosis] should be included, this issue is everywhere, let's say overdiagnosed, although you [the rest of the group] don't like the word" [Dois]  [Text] The potential for overdiagnosis was not mentioned explicitly, but a few GPs described the possibility of being diagnosed with and treated for a cancer that will not cause problems. [Smith]  [Text] Other aspects that  have been identified in the literature as necessary for the effective implementation of the IDM in the context of PSA counselling, such as possible anxiety during the waiting period for test results, the possibility of overdiagnosis  uncertainties about the benefits or  side effects of treatment are not addressed in the focus groups [Malli]. |

## *Circumstantial evidence that several participants have an accurate knowledge of overdiagnosis resulting from screening*

| **Finding** | **Studies contributing to this finding** | **Quote / Text** |
| --- | --- | --- |
| Circumstantial evidence that several participants have an accurate knowledge of overdiagnosis resulting from screening | Clements[5]  Dois[3]  Parker_ODx[6]  Pickles[7]  Smith[4]  Toledo-Chávarri[8]  Walters[9] | Quote: "... my usual line is, when old boys die an awful lot of them have been found to have prostate cancer that they had no idea was there and has never caused a jot of problems...and I try and explain that to them 'You may turn out to have the prostate cancer that is just going to sit there for thirty years and do nothing...' " [Clements]  [Text] Participants pointed out that, as controversial as the term may be, it is important to also explain the possibility of overdiagnosis that can be generated by mammography. This is understood as the identification of health problems that would not have caused harm to the individual. Its main causes relate to overdetection (identification of abnormalities that would not have caused harm) and overdefinition of disease (changing the boundaries for risk factors). This should be clearly communicated to women, when assessing their personal risk of breast cancer and the consequences of whether or not to be screened [Dois].  Quote: "Potentially treating them with serious treatments for a condition that maybe didn’t need to be found in the first place… "[Parker_ODx]  Quote: “There is a recognition that there are tumours found that are either frankly non-progressive or are likely to progress so slowly they don’t matter. I don’t think too many people would say, ‘Well that wouldn’t exist at all’. The argument is over how much and the scale of that.” (Expert #22, epidemiologist) [Parker_ODx]  Quote: “I wouldn’t go ahead, two steps ahead and discuss that they might find a cancer that they—that wouldn’t have killed them; I don’t go and—I don’t go there. I think, I mean, that’s sort of a urologist can do that” (GP7 [Pickles]  Quote:  -I just have to warn them… if a small cancer was found that small cancer may never have become symptomatic but once we’ve found it, in general, people are going to want to try to treat it and that could lead on to adverse effects. (M, >20 years’ experience, urban) [Smith]  - “So just explain the potential advantages and disadvantages… screening can pick things up, though it isn’t necessarily something you’d need to know, ‘cause you might live with it. And it never be a problem…” (P21) [Smith]  - “Though just because there’s little spots of cancer in the prostate doesn’t mean it’s ever going to be a problem, and doesn’t mean that necessarily needs any treatment. So we’ve known that about prostate cancer. But that really applies to the others to some extent.” P1 [Smith]  Quote: "It is true that all prevention programmes, and that has been demonstrated, have important side effects, and not all of them have the same benefits to compensate these side effects (…). We know that breast cancer screening reduces mortality, although there are some data that question it, and we also know that there is overdiagnosis, that many women are treated unnecessarily, which produces important psychological damage and a number of interventions in healthy women. Therefore we are producing harm. That is what data says after 20 years of studies" (primary care doctor). [Toledo-Chávarri]  [Text] The burden of ‘over diagnosis’ and false-positive results and the impact of treatment of screen detected cancers that would not necessarily have affected the woman during her natural lifetime were felt to be more pressing in the older age group. [Walters]  Quote: "If you have picked up a very small cancer in a very old lady who is frail and may shortly die from a heart attack then you are not really going to have done her any favours by taking her breast off... All you will have done is distress her enormously and caused a lot of pain." (Breast Surgeon) [Walters]  Quote: "Yes we may over diagnose and we do... But if we leave that low-grade DCIS there is no guarantee that it will not progress to high-grade and then invasive disease. So my feeling is that we will cause some psychological benefit by the tests that we do to a small proportion of women." (Radiologist) [Walters]  Quote: "We are looking for smaller and smaller lesions, we are picking up little bits of micro calcification, we are bringing women back for repeat biopsies... for the detection of smaller and smaller lesions that may never even have been invasive cancer. And even if they had been invasive cancer they might never have threatened their lives." (Radiologist) |

## *Overdiagnosis = when the biopsy already removed all the cancer*

| **Finding** | **Studies contributing to this finding** | **Quote / Text** |
| --- | --- | --- |
| Overdiagnosis = when the biopsy already removed all the cancer | Toledo-Chávarri[8] | Quote: "Sometimes a biopsy is done with thick needles in a lesion formed only by grouped microcalcification and incidentally it takes out all the tumour material. Indeed, when the patient arrives to the operating room and they made a resection of the area, then, they think that she does not have cancer at all, but no, it’s in the biopsy! If in the biopsy there is cancer from the morphological or immunohistochemical, there is cancer, and that woman, for better or worse, that this controversy appears or is maintained because someone can say “oh, I don’t have anything and when they removed that nothing appeared”, because a grouped microcalcification was removed and the tumour was all there" (oncologist). [Toledo-Chávarri] |

# Perception of overdiagnosis

## *Overdiagnosis is a devastating harm*

| **Finding** | **Studies contributing to this finding** | **Quote / Text** |
| --- | --- | --- |
|  | Parker_ODx[6]  Parker_values[10]  Pickles[7]  Walters[9] | [Text] Harm quantity was described in terms of the high number of overdiagnosed cases compared to the number of lives saved by screening. [Parker_ODx]  [Text] Harm quantity was described in terms of the high number of overdiagnosed cases compared to the number of lives saved by screening. Harm quality was discussed by highlighting the serious negative impact from each case of overdiagnosis, including both the psychological impact of a breast cancer diagnosis on a woman and her female relatives (for whom it has perceived risk implications), and the short and long term impact of unnecessary treatment on lifestyle and physical health. [Parker_ODx]  [Text] Expert A (#21 epidemiologist) saw breast-screening harm in terms of overdiagnosis and considered these harms to be substantial. This expert saw both avoiding harms and delivering benefits as being important, but because harms were, in their view, so large, was not supportive of the breast-screening programme. [Parker_values]  [Text] GPs considered underdiagnosis as well as overdiagnosis GPs discussed the harms of underdiagnosis (the missed opportunity to intervene in potentially preventable deaths) as much as those of overdiagnosis (the psychological and physical harms and ﬁnancial costs of unnecessary diagnosis and treatment). Since both harms are salient and serious, PSA testing decisions were described as a “balancing act” (GP21) or gamble. GPs reported the difﬁculties of needing to choose between potential harms (eg, incontinence and impotence) and the chance of saving lives. [Pickles]  [Text] These GPs believed the harms of overdiagnosis were too great to justify testing, “Even though we—in the long term you might save someone’s life, if you do an awful lot of harm along the way, it’s just not worth it” (GP18) [Pickles]  Quote: "If you have picked up a very small cancer in a very old lady who is frail and may shortly die from a heart attack then you are not really going to have done her any favours by taking her breast off... All you will have done is distress her enormously and caused a lot of pain." (Breast Surgeon) [Walters] |

## Overdiagnosis is a minor problem/necessary evil

| **Finding** | **Studies contributing to this finding** | **Quote / Text** |
| --- | --- | --- |
| Overdiagnosis is a minor problem | Parker_ODx[6] | [Text] Secondly, the number of overdiagnosed cases was considered low relative to the total number of breast cancers picked up through the program. [Parker_ODx]  Quote: “Harm is a term that’s been developed by academics, along academic lines… There’sa possibility ofover diagnosis … it’s not very much … you shouldn’t call that harmful.” (Expert #17, consumer advocate) [Parker_ODx]  [Text] Experts using this frame regarded overdiagnosis as a minor problem, for several reasons. Firstly, and most commonly, it was seen as an inevitable part of screening, particularly breast screening where cancer growth is variable and unpredictable. Secondly, the number of overdiagnosed cases was considered low relative to the total number of breast cancers picked up through the program. Finally, the harm associated with each overdiagnosed case was seen as low. This was justified in several ways: 1) individual women could not know whether or not their cancer was a case of overdiagnosis; 2) women1 (allegedly) disregarded the concept of overdiagnosis when considering treatment options; and 3) treatmen2t for small, low-grade cancers (ie those most likely to be cases of overdiagnosis) was viewed as relatively benign. [Parker_ODx]  [Text] Their central concern was not so much that overdiagnosis was mentioned, but that overdiagnosis was invariably (mis)represented as an important harm: “Harm is a term that’s been developed by academics, along academic lines… There’sa possibility of overdiagnosis … it’s not very much … you shouldn’t call that harmful.” (Expert #17, consumer advocate) [Parker_ODx]  [Text] Finally, the harm associated with each overdiagnosed case was seen as low. This was justified in several ways: 1) individual women could not know whether or not their cancer was a case of overdiagnosis; [Parker_ODx]  [Text] women (allegedly) disregarded the concept of overdiagnosis when considering treatment options; and treatment for small, low-grade cancers (ie those most likely to be cases of overdiagnosis) was viewed as relatively benign. [Parker_ODx]  [Text] treatment for small, low-grade cancers (ie those most likely to be cases of overdiagnosis) was viewed as relatively benign. [Parker_ODx] |
| Overdiagnosis is a necessary evil | Pickles[7]  Walters[9] | [Text] These GPs focused on cancer as life-threatening, and prostate cancer as a terrible death. They saw preventing death as the primary duty of the GP. This heightened their responsibility to do anything that may diagnose cancer early: “Because if you don’t overdiagnose, the alternative is to underdiagnose” (GP28). [Pickles]  [Text] Heuristic 1: GP preference to offer PSA testing to avoid underdiagnosis GPs employing heuristic 1 thought testing was necessary because there was a possibility it might prevent a man’s death. Overdiagnosis was perceived as (1) a natural consequence of PSA testing; (2) better than dying and (3) a justiﬁable source of harm (harms being a regrettable but necessary price of ‘cure’). [Pickles]  Quote: "Yes we may over diagnose and we do... But if we leave that low-grade DCIS there is no guarantee that it will not progress to high-grade and then invasive disease. So my feeling is that we will cause some psychological benefit by the tests that we do to a small proportion of women." (Radiologist) [Walters] |

## Overtreatment is the problem, not overdiagnosis

| **Finding** | **Studies contributing to this finding** | **Quote / Text** |
| --- | --- | --- |
| Overtreatment is the problem, not overdiagnosis | Parker_comm  Parker_ODx[6]  Pickles[7] | Quote: “I always like to think [the harm] is not necessarily overdiagnosis but overtreatment … So don’t stop yourself from actually being diagnosed but then when you get the information, it’s what you do with it. And the patient needs to be very well informed around what your risks are if there’s no treatment versus the treatment (Expert #6, consumer advocate) [Parker_comm]  [Text] In this frame, both mortality benefit and harm avoidance were valued. Thus appropriate solutions in this frame maintained current screening parameters, and only altered downstream elements. Experts presented a range of solutions including: regular pathology updates on diagnostic criteria and thresholds; research into better prognostic tools (such as biological markers of aggression); development of more targeted / less harmful therapies, research into less aggressive treatment regimes for low-risk lesions; and patient-centred care for women with borderline lesions, relying on correlation between clinical, radiological and pathological findings to make a diagnosis and plan the management, rather than following set guidelines. [Parker_ODx]  [Text] The final frame through which overdiagnosis was understood purposefully separated the treatment process from the screening process, and presented the problem as arising from treatment decisions. [Parker_ODx]  # [Text] Experts presented a range of solutions including: regular pathology updates on diagnostic criteria and thresholds; research into better prognostic tools (such as biological markers of aggression); development of more targeted / less harmful therapies, research into less aggressive treatment regimes for low-risk lesions; [Parker_ODx]  Quote: “I don’t really believe in overdiagnosis as such. I mean, I think there’s over treatment … Finding it is not the issue. Treating – how it’s treated is the issue, as I see it.” (Expert #9, clinician and provider) [Parker_ODx]  [Text] Some of these GPs thought decisions about postdiagnosis management (eg, active surveillance) could limit the harms of potential overdiagnosis. This allowed them to deﬁne testing without invasive procedures as inconsequential: “it’s not terribly onerous to have a blood test every six months” (GP3) [Pickles] |

## Overdiagnosis is a normal consequence of screening

| **Finding** | **Studies contributing to this finding** | **Quote / Text** |
| --- | --- | --- |
| Overdiagnosis is a normal consequence of screening | Parker_comm[11]  Parker_ODx[6]  Toledo-Chávarri[8] | [Text] Some experts added to this by asserting that overdiagnosis was not a harm, rather that the diagnosis of small cancers was exactly what the screening program was intended to do in order to reduce breast cancer mortality and morbidity. [Parker_comm]  [Text] Firstly, and most commonly, it was seen as an inevitable part of screening, particularly breast screening where cancer growth is variable and unpredictable. [Parker_ODx]  [Text] For these professionals, overtreatment was a consequence of the interventions, which are necessary to diagnose cancer, and their effects on women were considered minimal. [Toledo-Chávarri] |

## Overdiagnosis is not a harm, it may even be beneficial

| **Finding** | **Studies contributing to this finding** | **Quote / Text** |
| --- | --- | --- |
| Overdiagnosis is not a harm, it may even be beneficial | Parker_comm[11]  Parker_ODx[6]  Parker_values[10]  Pickles[7] | [Text] Some stated that the concept of overdiagnosis being a harm was based on opinion, rather than fact and therefore did not count as information. [Parker_comm]  Quote “Harm is a term that’s been developed by academics, along academic lines… [Overdiagnosis is not] women’s definition of harm” (Expert #24, consumer advocate) [Parker_comm]  Quote: “[Information on overdiagnosis] is a bit of a worry, because of the way it’s presented and interpreted … Women aren’t being harmed by breast screening and society isn’t being harmed by breast screening… It comes from the epidemiologists, who are quite far removed from actually having breast cancer or treating it - they’re looking at populations and then they take it upon themselves … to actually put their personal view as to what this might be doing to women, what harm it might be doing, which is very unscientific. **Early diagnosis, breast screening, leads to more of your diagnoses and overtreatment and that’s [not] a harm, it’s a value…** That bit of information [about the harms of overdiagnosis] is opinion interpretation… You have to be very careful what information you do give [women] and that you’re not giving them a set of facts that’s been interpreted by one kind of view (Expert #13, consumer advocate) [Parker_comm]  [Text] In addition to the lack of harm, the frame highlighted possible benefits from overdiagnosis. Although, by definition, an overdiagnosed cancer will not itself threaten a woman’s life, experts suggested that as the woman would be at increased risk of a second breast cancer she would benefit from being identified and treated with tamoxifen. [Parker_ODx]  [Text] A third, less widely expressed view about harms concentrated on women’s experience of the screening process. This view was held by all three consumer advocates and one researcher, who described harm in terms of minor physical discomfort and inconvenience, and denied that overdiagnosis or false positives caused harm: Women aren’t being harmed by breast screening and society isn’t being harmed by breast screening. It’s … a little mindset that has developed. (#13 consumer advocate)[Parker_values]  [Text] GPs valued the knowledge produced by the [PSA]test which can reassure patients and the GP. “I believe there is no case for saying you shouldn’t take PSAs…how can knowledge not be a good thing?” (GP29) [Pickles] |

# Screening policy

## Avoid screening (a.o. because of overdiagnosis)

| **Finding** | **Studies contributing to this finding** | **Quote / Text** |
| --- | --- | --- |
| Avoid screening (a.o. because of overdiagnosis) | Parker_ODx[6]  Parker_values[10]  Pickles[7] | [Text] Reduce overdiagnosis either by performing targeted screening or by reducing screening overall. Experts who were more sceptical about the benefits accruing from breast screening preferred a more extreme solution: reducing overdiagnosis by decreasing overall breast screening participation. [Parker_ODx]  [Text] the removal of governmental promotions and personalised screening invitations.[Parker_ODx]  [Text] they assumed that cessation of public funding for the program was politically unlikely [Parker_ODx]  This framing of overdiagnosis as a serious problem was grounded in a strong commitment to avoiding harm in any public health program. [Parker_ODx]  # [Reduce overdiagnosis either by performing targeted screening or by reducing screening overall [Parker_ODx]  [Text] Expert A (#21 epidemiologist) saw breast-screening harm in terms of overdiagnosis and considered these harms to be substantial. This expert saw both avoiding harms and delivering benefits as being important, but because harms were, in their view, so large, was not supportive of the breast-screening programme. [Parker_values]  [Text] A smaller number of experts discussed conﬂicting values in terms of avoiding harms and delivering beneﬁts. Their view about the relative importance of these two values had practical implications for whether or not they supported breast screening: those who prioritised avoiding harm were less likely to support screening than those who prioritised delivering beneﬁts. [Parker_values]  [Text] Some GPs were strongly oriented to avoiding overdiagnosis, and so tried to test as little as possible. This group of GPs emphasised the harms of PSA testing (including overdiagnosis) when advising their patients; and said many patients chose not to be tested following discussion. These GPs, who fully explain overdiagnosis, described themselves as “taking the risk of doing the hard work, hard yards” (GP23). [Pickles]  [Text] These GPs believed the harms of overdiagnosis were too great to justify testing, “Even though we—in the long term you might save someone’s life, if you do an awful lot of harm along the way, it’s just not worth it” (GP18) [Pickles]  [Text] GPs employing heuristic 2 preferred not to conduct PSA testing. Their primary justiﬁcation was preventing harms caused by overdiagnosis. However, while they would try to talk patients out of having the test, they would never refuse a PSA test. These GPs also recognised that PSA testing has saved lives; “we know that happens. The problem is, it just doesn’t happen often enough to balance out…all the damage that we do” (GP17). [Pickles]  [Text] GPs considered underdiagnosis as well as overdiagnosis GPs discussed the harms of underdiagnosis (the missed opportunity to intervene in potentially preventable deaths) as much as those of overdiagnosis (the psychological and physical harms and ﬁnancial costs of unnecessary diagnosis and treatment). Since both harms are salient and serious, PSA testing decisions were described as a “balancing act” (GP21) or gamble. GPs reported the difﬁculties of needing to choose between potential harms (eg, incontinence and impotence) and the chance of saving lives. [Pickles]  [Text] Prefer not to test or will test only under duress because of uncertain benefit and potential harms caused by overdiagnosis. GP tries to minimise the likelihood of overdiagnosis by minimising PSA testing. [Pickles]  # [GPs considered underdiagnosis as well as overdiagnosis GPs discussed the harms of underdiagnosis (the missed opportunity to intervene in potentially preventable deaths) as much as those of overdiagnosis (the psychological and physical harms and ﬁnancial costs of unnecessary diagnosis and treatment). Since both harms are salient and serious, PSA testing decisions were described as a “balancing act” (GP21) or gamble. GPs reported the difﬁculties of needing to choose between potential harms (eg, incontinence and impotence) and the chance of saving lives. [Pickles]  # [Prefer not to test or will test only under duress because of uncertain benefit and potential harms caused by overdiagnosis. GP tries to minimise the likelihood of overdiagnosis by minimising PSA testing. [Pickles] |

### Mental burden

| **Finding** | **Studies contributing to this finding** | **Quote / Text** |
| --- | --- | --- |
|  | Pickles[7] | Testing decisions were described as a personal burden Uncertainty about PSA testing created a ‘personal burden’ for some GPs; they felt personally responsible for the consequences of their PSA testing approach, and experienced guilt and self-blame as a result. [Pickles]  GPs found it hard knowing some cancers would be missed because of their decision not to test: for example, one described this as “a burden that I carry” (GP8). GPs most concerned about overdiagnosis experienced the highest levels of personal burden because, although relatively rare, death as the potential consequence of not testing was seen as the worst possible outcome. [Pickles]  Heavy personal burden reported. Many GPs carried the burden of hoping they had protected their patient from the harms of overdiagnosis while witnessing prostate cancer death. “I’m fully aware of the fact that prostate cancer kills my patients and that by not testing for it, I won’t find it…it’s an incredibly difficult situation” (GP8). GPs recalled cases of patients who had suffered with aggressive/ metastatic cancers and spoke of how hard it is to see men suffering (from potentially missed cancers) but to continue to practise according to their own values, the evidence and recommendations. “But I did what you were meant to do and that’s what’s going to happen sometimes, so it’s just hard to take” (GP27) [Pickles]  “I worry that there are men, young men who probably will get prostate cancer and die of it because I’m not doing enough screening, but I’m not prepared to, to not follow the evidence and I think that the evidence says you don’t do it” (GP8). However, it was extremely challenging for GPs to balance guidelines against anecdotal experiences; “even though you know the statistics, you are influenced by what you are dealing with at the time…if you hear the story instead of just the statistics, it makes a lot more powerful a case” (GP27) [Pickles] |

## Keep offering screening and support informed decision making (with a.o. comprehensive info about overdiagnosis)

| **Finding** | **Studies contributing to this finding** | **Quote / Text** |
| --- | --- | --- |
| Support informed decision making (with a.o. comprehensive info about overdiagnosis) | Gimenez[1]  Parker_comm[11]  Parker_ODx[6]  Pickles[7]  Toledo-Chávarri[8] | Some GPs wanted to avoid a paternalistic attitude. They preferred to provide full information, including on risks without fear of reducing screening participation [1].  “This is not straightforwardly a good thing. There are some downsides and while we don’t necessarily think the downsides are such that you shouldn’t be doing it, at the very least, we should be telling women about this so that they can make an informed decision.” Parker_comm  “[We should let] women know in an intelligent way about this complex topic so that they can be fully informed and make an informed choice … you don’t want to just say, trust me I’m a doctor” (Expert #22, clinician & provider) [Parker_comm]  “There’s actually a lot of evidence of harm here. If I look at it carefully, then you think oh, the benefits and the harms are much more finely balanced than I had actually appreciated, then a persuasion campaign is just indefensible.” (Expert #27, epidemiologist) [Parker_comm]  [Text] Experts discussed both overdiagnosis harms and mortality benefits accruing from breast screening. They suggested that while each are likely to be important to women, current estimates about their rates meant that harms and benefits were closely balanced; [Parker_ODx]  [Text] Some experts discussed seeking public assistance with decision making at the policy level, using a deliberative process such as a citizens’ jury to make a ruling about the balance between benefits and harms: “I believe that for a lot ofscreening things there should be a community jury. There are some things that are obvious, that we can just proceed with them, but other things where there’s a balance between the benefits and harms, I think we need some sort ofdeliberative democracy process.” (Expert #21, researcher NOS) [Parker_ODx]  [Text] It is impossible for experts to definitively compare harms & benefits because they are qualitatively different. It is not clear how to compare the harms & benefits of breast screening [Parker_ODx]  [Text] Frame 5: balancing harms and benefits is a personal matter “Descriptively they’re quite different … I don’t think there is any formula for the balance… It’s very subjective of the balance of disparate outcomes.” (Expert #20, clinician) [Parker_ODx]  Others spoke of more explicit attempts to achieve in- formed consumer decision making, encouraging women to consider the net value of screening for themselves as individuals. They suggested screening participation decisions should be based on women’s personal priorities rather than potentially coercive input from screening providers. [Parker_ODx]  Experts explained that since individual attitudes to harms and benefits would determine what was perceived as the net outcome of screening, the process of decision making needed consumer input: it was insufficient to rely on pre-determined program values or system priorities. The frame encompassed two possible solutions. Some experts discussed seeking public assistance with decision making at the policy level, using a deliberative process such as a citizens’ jury to make a ruling about the balance between benefits and harms. Others spoke of more explicit attempts to achieve informed consumer decision making, encouraging women to consider the net value of screening for themselves as individuals. They suggested screening participation decisions should be based on women’s personal priorities rather than potentially coercive input from screening providers [Parker_ODx]  [Text] They tended to consider decisions about PSA testing as neither right nor wrong and so could be swayed either way depending on the patient and their needs. [Pickles]  The personal burden experienced by these GPs was minimal as in most consultations the burden of decision making was shared with their patients. GPs sought to reach a mutual understanding of PSA testing if they thought the patient was able to understand the information required, and shared the responsibility of decisions and outcomes of the consultation with the individual man. [Pickles]  Quote: "We come from a perception that the intervention was very beneficial, once we have had more data and information, we have seen that the benefitharm balance is much narrower than it was thought, therefore it is necessary to give more information so women can decide (epidemiologist)." [Toledo-Chávarri]  [Text] On an ethical level, they posited that it is imperative to inform people about the available evidence because these data change the balance between the benefits and harms. [Toledo-Chávarri] |

## Adapt screening (make it more targeted, reduce downstream treatment harms)

| **Finding** | **Studies contributing to this finding** | **Quote / Text** |
| --- | --- | --- |
| Adapt screening (make it more targeted, reduce downstream treatment harms) | Gimenez[1]  Parker_ODx[6]  Pickles[7] | [Text] Certains MG imaginaient que le dépistage organisé pourrait laisser place à un dépistage individuel, ciblé, dans lequel le MG aurait un rôle prépondérant. [Gimenez]  [Text] Reduce overdiagnosis either by performing targeted screening or by reducing screening overall. Experts who were enthusiastic about the potential benefits of screening suggested reducing overdiagnosis through a targeted, personalised screening program, matching recommended screening frequency to breast cancer risk as determined by factors such as breast density. This would enable the population to simultaneously retain benefits of screening and reduce harms. [Parker_ODx]  [Text] Thus appropriate solutions in this frame maintained current screening parameters, and only altered downstream elements. While it is important that screening continues to save lives, we should seek ways to reduce harms from unnecessary (over)treatment [Parker_ODx]  [Text] Some of these GPs thought decisions about postdiagnosis management (eg, active surveillance) could limit the harms of potential overdiagnosis. This allowed them to deﬁne testing without invasive procedures as inconsequential: “it’s not terribly onerous to have a blood test every six months” (GP3) [Pickles] |

## Offer screening and encourage people (a.o. because overdiagnosis is not relevant)

| **Finding** | **Studies contributing to this finding** | **Quote / Text** |
| --- | --- | --- |
| Offer screening and encourage people (a.o. because overdiagnosis is not relevant) | Parker_ODx[6]  Parker_values[10]  Pickles[7]  Toledo-Chávarri[8] | [Quote: Breast screening commentators should give priority to delivering health benefits (saving lives) The frame delivers a choice between life and overdiagnosis: “saving a life is more important than the harm that’s caused in damaging normal breasts.” (Expert #3, clinician). [Parker_ODx]  [Text] This frame centres on the negative publicity generated by overdiagnosis discussions and the decrease in breast screening participation that might ensue. Underlying this concern is a firm belief in the net benefit of breast screening and a strong desire to have women avail themselves of life-saving opportunities. [Parker_ODx]  In this frame, appropriate solutions focussed on preventing a fall in participation rates. They included: avoiding any implication that overdiagnosis is a harm; keeping discussions confined to academic circles; and informing women about overdiagnosis only when attendance is secured (such as at the point of mammogram or after diagnosis). [Parker_ODx]  [Text] A smaller number of experts discussed conﬂicting values in terms of avoiding harms and delivering beneﬁts. Their view about the relative importance of these two values had practical implications for whether or not they supported breast screening: those who prioritised avoiding harm were less likely to support screening than those who prioritised delivering beneﬁts. [Parker_values]  [Text] Heuristic 1: GP preference to offer PSA testing to avoid underdiagnosis GPs employing heuristic 1 thought testing was necessary because there was a possibility it might prevent a man’s death. Overdiagnosis was perceived as (1) a natural consequence of PSA testing; (2) better than dying and (3) a justiﬁable source of harm (harms being a regrettable but necessary price of ‘cure’). [Pickles]  [Text] These GPs focused on cancer as life-threatening, and prostate cancer as a terrible death. They saw preventing death as the primary duty of the GP. This heightened their responsibility to do anything that may diagnose cancer early: “Because if you don’t overdiagnose, the alternative is to underdiagnose” (GP28). [Pickles]  [Text] Mostly test. Testing is perceived to be an absolute obligation. These GPs feel their primary duty is to prevent prostate cancer death, and save men’s lives wherever possible. “To not screen somebody, I don’t know, it seems cruel, it’s cruel and irresponsible… to not at least make an attempt to avoid the misery of a person getting prostate cancer, to me, seems unbelievably cruel” (GP29). “We have to diagnose them if they have a problem. What if it couldn’t wait? How would you know it won’t affect them?” (GP11) [Pickles]  [Text] Some professionals, both clinicians and screening programme workers, positioned themselves against providing information about the screening harms. They argued that there exists enough evidence on the benefits of the programme and therefore, screening is not ethically questionable. This evidence guides the screening programmes’ design and does not allow for any decision- making process among women. [Toledo-Chávarri] |

# Preparedness to provide information about ODx

## Provide comprehensive information to discourage screening and avoid harm

| **Finding** | **Studies contributing to this finding** | **Quote / Text** |
| --- | --- | --- |
| Provide comprehensive information to discourage screening and avoid harm | (Parker_comm[11])  (Smith[4])  Pickles[7] | # [Quote: “They need to be provided with adequate information to know what they are signing up for. If they are coming to screening they need to know.” (Expert #33, clinician & provider) [Parker_comm]  # [These GPs, who fully explain overdiagnosis, described themselves as “taking the risk of doing the hard work, hard yards” (GP23). They resisted medicolegal fears by engaging in detailed discussions of beneﬁts and harms, and felt covered from legal prosecution by the Royal Australian College of General Practitioners (RACGP) guidelines. [Pickles]  # [[Text] GPs inclined to talk about overdiagnosis and emphasise the harms of PSA testing when giving advice. Most of these GPs try to talk patients out of having the test, and many had patients who did not go ahead with a PSA test following discussion [Pickles]  # [[Text] Some GPs were strongly oriented to avoiding overdiagnosis, and so tried to test as little as possible. This group of GPs emphasised the harms of PSA testing (including overdiagnosis) when advising their patients; and said many patients chose not to be tested following discussion. These GPs, who fully explain overdiagnosis, described themselves as “taking the risk of doing the hard work, hard yards” (GP23). [Pickles]  [Text] The potential for overdiagnosis was not mentioned explicitly, but a few GPs described the possibility of being diagnosed with and treated for a cancer that will not cause problems. [Smith]  Quote:  -I just have to warn them… if a small cancer was found that small cancer may never have become symptomatic but once we’ve found it, in general, people are going to want to try to treat it and that could lead on to adverse effects. (M, >20 years’ experience, urban) [Smith]  - “So just explain the potential advantages and disadvantages… screening can pick things up, though it isn’t necessarily something you’d need to know, ‘cause you might live with it. And it never be a problem…” (P21) [Smith]  - “Though just because there’s little spots of cancer in the prostate doesn’t mean it’s ever going to be a problem, and doesn’t mean that necessarily needs any treatment. So we’ve known that about prostate cancer. But that really applies to the others to some extent.” P1 [Smith] |

## Provide comprehensive information to support informed decision making about screening

| Provide comprehensive information to support informed decision making about screening | Dois[3]  Gimenez[1]  Parker_comm[11]  Parker_ODx[6]  Parker_values[10]  Pickles[7]  (Smith[4])  Toledo-Chávarri[8] | [Text] Participants pointed out that, as controversial as the term may be, it is important to also explain the possibility of overdiagnosis that can be generated by mammography.[Dois]  Quote: "But I think it [overdiagnosis] should be included, this issue is everywhere, let's say overdiagnosed, although you [the rest of the group] don't like the word" [Dois]  [Text] Some GPs wanted to avoid a paternalistic attitude. They preferred to provide full information, including on risks without fear of reducing screening participation [Gimenez]  [Text] Full information: Experts who preferred providing full information to women advocated for the following content in consumer communications: • Detailed information about mortality benefit, false positives & overdiagnosis associated with breast screening  • Numerical / pictorial comparison of chances of deriving benefit & being  overdiagnosed [Parker_comm]  [Text] Contrary to this position, a smaller group of experts advocated full information about both benefits and harms of breast screening. They particularly wanted consumers to be provided with understandable data about overdiagnosis, including readily comparable information on chances of mortality benefit versus overdiagnosis. [Parker_comm]  # They suggested that independent consumer decision making was particularly important in breast screening because of the close balance between benefits and harms, and what experts saw as the individual nature of the benefits. [Parker_comm]  [Quote “This is not straightforwardly a good thing. There are some downsides and while we don’t necessarily think the downsides are such that you shouldn’t be doing it, at the very least, we should be telling women about this so that they can make an informed decision.” (Expert #20, epidemiologist) [Parker_comm]  # [Quote: “They need to be provided with adequate information to know what they are signing up for. If they are coming to screening they need to know.” (Expert #33, clinician & provider) [Parker_comm]  [Text] Overall more experts preferred guiding women to be screened, and overall more experts preferred that full information be provided. [Parker_comm]  # [Quote: “[We should let] women know in an intelligent way about this complex topic so that they can be fully informed and make an informed choice … you don’t want to just say, trust me I’m a doctor” (Expert #22, clinician & provider) [Parker_comm]]  Experts who advocated against guidance were worried about overdiagnosis harms and were enthusiastic about enabling individual consumers to make their own decisions about health. [Parker_comm]  [Text] Experts who preferred full information argued that consumers should be informed about what they were being asked to do. In particular these experts claimed that full information on overdiagnosis was important for its instrumental role in informed consumer decision making. [Parker_comm]  [Text] They suggested that independent consumer decision making was particularly important in breast screening because of the close balance between benefits and harms, and what experts saw as the individual nature of the benefits. [Parker_comm]  - Quote “There’s actually a lot of evidence of harm here. If I look at it carefully, then you think oh, the benefits and the harms are much more finely balanced than I had actually appreciated, then a persuasion campaign is just indefensible.” (Expert #27, epidemiologist) [Parker_comm]  [Text] This frame centres on the lack of communication about overdiagnosis from screening providers to women. Experts acknowledged that while some women prefer a simple advisory message about breast screening, others want an informed decision making process, with the readily available and easily-understood information [Parker_ODx]  [Text] Frame 3: don’t hide the overdiagnosis problem from women “We should absolutely tell people, ‘These are the benefits, these are the harms’; and some people say that public health benefits should be what we are aiming for, but for me I think you absolutely cannot compromise on telling people. It’s just not something I’m prepared to do.” (Expert #23, researcher NOS) [Parker_ODx]  [Text] The solution was to make information about overdiagnosis available to women, despite the inherent complexities in the topic and the tension with trying to encourage participation: “I agree with you that the experts can’t agree and how do you talk to women about it, and it is a very complex area and hard to talk about, but clearly an important issue in the context ofscreening… I think you have to share with women your uncertainty.” (Expert #25, epidemiologist) [Parker_ODx]  [Text] The current lack of communication about overdiagnosis was presented as a deliberate strategy by screening providers to avoid risking a decline in participation. In this frame, informed choice was an absolute right for individual women, taking priority over the delivery of population health benefits. [Parker_ODx]  [Text] This frame accommodated a variety of solutions ranging from detailed publicising of overdiagnosis information in every screening pamphlet and advertisement, to making detail of possible harms from screening available upon request. In this frame provision of information could coexist alongside government promotion of screening. [Parker_ODx]  and some people say that public health benefits should be what we are aiming for, but for me I think you absolutely cannot compromise on telling people. It’s just not something I’m prepared to do.” (Expert #23, researcher NOS) [Parker_ODx]  The solution was to make information about overdiag-nosis available to women, despite the inherent complexities in the topic and the tension with trying to encourage participation: [Parker_ODx]  “I agree with you that the experts can’t agree and how do you talk to women about it, and it is a very complex area and hard to talk about, but clearly an important issue in the context ofscreening… I think you have to share with women your uncertainty.” (Expert #25, epidemiologist) [Parker_ODx]  [Text] Experts expressed differing versions of what respecting autonomy means in breast screening. The dominant view was that respecting autonomy is about providing comprehensive information to women who are offered breast screening. [Parker_values]  [Text] Those who prioritised autonomy were in favour of providing more comprehensive information and encouraging informed choice. [Parker_values]  [Text] The potential for overdiagnosis was not mentioned explicitly, but a few GPs described the possibility of being diagnosed with and treated for a cancer that will not cause problems. [Smith]  Quote:  -I just have to warn them… if a small cancer was found that small cancer may never have become symptomatic but once we’ve found it, in general, people are going to want to try to treat it and that could lead on to adverse effects. (M, >20 years’ experience, urban) [Smith]  - “So just explain the potential advantages and disadvantages… screening can pick things up, though it isn’t necessarily something you’d need to know, ‘cause you might live with it. And it never be a problem…” (P21) [Smith]  - “Though just because there’s little spots of cancer in the prostate doesn’t mean it’s ever going to be a problem, and doesn’t mean that necessarily needs any treatment. So we’ve known that about prostate cancer. But that really applies to the others to some extent.” P1 [Smith]  [Text] The personal burden experienced by these GPs was minimal as in most consultations the burden of decision-making was shared with their patients. GPs sought to reach a mutual understanding of PSA testing if they thought the patient was able to understand the information required, and shared the responsibility of decisions and outcomes of the consultation with the individual man.[Pickles]  [Text] No fear of medicolegal Q. Perceived themselves to be covered by the guidelines of their medical college. They engaged patients in detailed discussions about potential harms and resisted medicolegal fears. “If I did that…I think I would be a more paternalistic doctor who ordered a lot more tests. And I don’t see that would be good medicine. I think it would do more harm to more people for practicing defensively like that” (GP30) [Pickles]  Quote: "Let’s see, I really think that (…) we all have worked on these issues, no matter how much - or how little, but we have worked on them and we are sliding in: It is no necessary shared decision- making in that issue. Don’t do it. That is what I am perceiving (from the discussion into this group). As we know breast cancer screening is right women don’t have to decide (…). We do not want a shared decision; that is what I understand (…). In addition not giving information because if we give it, considering that there are dubious studies, participation will go down. That’s the conclusion I don’t agree (primary care doctor)". [Toledo-Chávarri]  Quote: "We come from a perception that the intervention was very beneficial, once we have had more data and information, we have seen that the benefit harm balance is much narrower than it was thought, therefore it is necessary to give more information so women can decide (epidemiologist)." [Toledo-Chávarri]  [Text] On an ethical level, they posited that it is imperative to inform people about the available evidence because these data change the balance between the benefits and harms. [Toledo-Chávarri]  [Text] Those who think that the decision should be shared between professionals and women. In this case, it is necessary to establish a dialogue between women and health professionals to discuss risks and benefits of screening. The majority of professionals favoured providing information to women[Toledo-Chávarri] |
| --- | --- | --- |

## Provide limited and reassuring information about overdiagnosis to avoid confusing people

| Provide limited and reassuring information about overdiagnosis to avoid confusing people | Pickles[7]  Toledo-Chávarri[8] | They suggested overdiagnosis information should be presented briefly along the lines of, “some of the things that we are going to be treating you for may not progress.” (Expert #33, clinician and provider) These experts  proposed that further information could be made available for those who wanted it. [Parker_ODx]  Quote: "and informing women about overdiagnosis only when attendance is secured (such as at the point of mammogram or after diagnosis)". [Parker_ODx]  These GPs approached communication in several different ways. Some made their own decision about the ‘right’ approach for each particular patient, and advised that patient accordingly. This could include not discussing overdiagnosis at all, on the grounds that it was irresponsible to expect patients to understand complex information; “if you start going down that road and— and to what end?” (GP7). [Pickles]  [Text] They were in favour of informed participation and SDM, although some specified that their support was limited to situations that did not generate fear or confusion. [Toledo-Chávarri] |
| --- | --- | --- |

## Provide limited to no information about overdiagnosis to avoid reducing participation and save lives

| Provide limited to no information about overdiagnosis to avoid reducing participation and save lives | Gimenez[1]  Parker_ODx[6]  Parker_values[10]  Pickles[7]  Toledo-Chávarri[8] | À l'inverse, d'autres MG estimaient qu'évoquer le surdiagnostic diminuerait la participation au dépistage ou sera it source de confusion pour les patientes.[Gimenez]  Quote: "and informing women about overdiagnosis only when attendance is secured (such as at the point of mammogram or after diagnosis)". [Parker_ODx]  [Text] There was also moral condemnation of the particular impact that negative publicity has upon disadvantaged women. This group was presented as being particularly likely to be confused by public debates, and vulnerable to screening disengagement: “There’s probably people in the [suburbs of lower socioeconomic status] who stop going to screening. Because they’re not as sophisticated … and they come from non-English speaking backgrounds. The message they get is that screening is not needed… It’s okay if you’re in the [suburbs of higher socioeconomic status] because you’ll keep coming anyway.” (Expert #29, clinician) [Parker_ODx]  Those who prioritised delivering beneﬁts, for example, preferred to limit breast-screening information in order to avoid frightening women away. [Parker_values]  GPs with this practice orientation advised men to have a PSA, emphasising beneﬁts of early detection, and did not discuss overdiagnosis. [Pickles]  GPs inclined not to talk about overdiagnosis. The information they provide is “next to none” (GP9). Communication style characterised by advising men to have a PSA with emphasis on benefits of early detection [Pickles]  Quote: "Let’s see, I really think that (…) we all have worked on these issues, no matter how much - or how little, but we have worked on them and we are sliding in: It is no necessary shared decision- making in that issue. Don’t do it. That is what I am perceiving (from the discussion into this group). As we know breast cancer screening is right women don’t have to decide (…). We do not want a shared decision; that is what I understand (…). In addition not giving information because if we give it, considering that there are dubious studies, participation will go down. That’s the conclusion I don’t agree (primary care doctor)". [Toledo-Chávarri]  [Text] Some professionals, both clinicians and screening programme workers, positioned themselves against providing information about the screening harms. They argued that there exists enough evidence on the benefits of the programme and therefore, screening is not ethically questionable. This evidence guides the screening programmes’ design and does not allow for any decision- making process among women. [Toledo-Chávarri]  Quote: There is enough evidence about benefits of the programme and, therefore, they can’t be debated nor ethically questioned. Its role is to guide screening programmes planning and not women’s decisions. Those are things that can’t be debated, as there are some standards that have already been established by scientific evidence and are not debatable (screening programme professional). [Toledo-Chávarri]  [Text] Those who consider that screening is beneficial for women’s health, and therefore it is not necessary that women participate in the decision-making process because it is clear that they should participate in screening. [Toledo-Chávarri]  [Text] Those who believe that SDM is not possible because the rationale for the screening programmes is the evidence of benefit. [Toledo-Chávarri]  [Text] The decision is “Yes, participate” as it is recommended by the programme because there is no margin for SDM within the current health system. [Toledo-Chávarri] |
| --- | --- | --- |

## Informing about overdiagnosis is very difficult, and complex, perhaps impossible

| Informing about overdiagnosis is very difficult, and complex, perhaps impossible | Parker_ODx[6]  Pickles[7] | [Text] Some experts used this frame with the view that in- formed choice was an unattainable goal, because overdiagnosis in breast screening is just so complex: [Parker_ODx]  [Text] The solution was to make information about overdiag- nosis available to women, despite the inherent complexities in the topic and the tension with trying to encourage participation: “I agree with you that the experts can’t agree and how do you talk to women about it, and it is a very complex area and hard to talk about, but clearly an important issue in the context ofscreening… I think you have to share with women your uncertainty.” (Expert #25, epidemiologist) [Parker_ODx]  Other GPs tailored their discussion about overdiagnosis to the needs of the individual patient, their perceived level of understanding and time pressures: “it gets more complicated depending on how interested the person is” (GP4). Thus, the GP’s communication depended entirely on the individual patient in front of them. [Pickles]  GPs who approached PSA testing case by case gener- ally agreed that overdiagnosis statistics do not, or cannot, apply to individuals; “those like statistical issues don’t apply to the individual…because…they make their decisions on a set of complex, but perhaps irrational basis, you know, anxiety and…” (GP7) Accordingly, they tailored their testing and patient communication but expressed some difﬁculties in translating population based information to individuals [Pickles] |
| --- | --- | --- |

## Talking about overdiagnosis is only relevant after a disease would have been diagnosed

| Talking about overdiagnosis is only relevant after a disease would have been diagnosed | Clements[5]  Pickles[7] | [Text] Some of these GPs felt explaining overdiagnosis was the responsibility of urologists, [Pickles]  Quote "I feel I'm in second line for that (discussion of range of prostate cancers) because if they go ahead and have the biopsy, say they have the PSA test and it's positive then you have to, you are obliged to refer ... the urologists are obliged then to investigate further.....so it's very much their stance isn't it?" [Clements] |
| --- | --- | --- |

## Do not think and talk about overdiagnosis

| Do not think and talk about overdiagnosis | Clements[5]  Dois[3]  Malli[2]  Pickles[7]  Smith[4] | [Text] Some GPs did not think about underdiagnosis or overdiagnosis at all. [Pickles]  [Text] Heuristic 4: GP preference to avoid thinking about underdiagnosis or overdiagnosis GPs not thinking about underdiagnosis or overdiagnosis did not have a preference or priority for avoiding one harm over another. For these GPs, the PSA test was considered just another form of routine screening and underdiagnosis or overdiagnosis was not an issue of concern. [Pickles]  [Text] For some, it was easiest to just do the PSA test with no explanation at all [Pickles]  [Text] Other aspects that have been identified in the literature as necessary for the effective implementation of the IDM in the context of PSA counselling, such as possible anxiety during the waiting period for test results, the possibility of overdiagnosis uncertainties about the benefits or side effects of treatment are not addressed in the focus groups [Malli]  [Text] While less frequently discussed than the previous key  points, a number of GPs said they would discuss the variable  nature of prostate cancer, with an emphasis on the  possibility of identifying indolent cancers which may  never cause a problem [Clements] |
| --- | --- | --- |

1. Gimenez L, Janczewski A. Representation of overdiagnosis in breast cancer screening among general practitioners: a qualitative study by focus groups. Exercer-La Revue Francophone De Medecine Generale. 2018(139):18-9.

2. Malli G. [Early detection of prostate cancer by PSA testing: the results of a qualitative study on barriers caused by physicians in Austria implementing informed decision making]. Gesundheitswesen (Bundesverband der Arzte des Offentlichen Gesundheitsdienstes (Germany)). 2013;75(1):22-8.

3. Dois A, Bravo P, Fernández-González L, Uribe C. [Experts' views on the communication of risks and benefits of mammography to detect breast cancer]. Rev Med Chil. 2021;149(2):196-202.

4. Smith J, Dodd RH, Wallis KA, Naganathan V, Cvejic E, Jansen J, et al. General practitioners' views and experiences of communicating with older people about cancer screening: a qualitative study. Fam Pract. 2022.

5. Clements A, Watson E, Rai T, Bukach C, Shine B, Austoker J. The PSA testing dilemma: GPs' reports of consultations with asymptomatic men: a qualitative study. BMC Fam Pract. 2007;8:1-7.

6. Parker LM, Rychetnik L, Carter S. Framing overdiagnosis in breast screening: a qualitative study with Australian experts. BMC Cancer. 2015;15:606.

7. Pickles K, Carter SM, Rychetnik L. Doctors' approaches to PSA testing and overdiagnosis in primary healthcare: a qualitative study. BMJ open. 2015;5(3):e006367.

8. Toledo-Chávarri A, Rué M, Codern-Bové N, Carles-Lavila M, Perestelo-Pérez L, Pérez-Lacasta MJ, et al. A qualitative study on a decision aid for breast cancer screening: Views from women and health professionals. European Journal of Cancer Care. 2017;26(3).

9. Walters SJ, Winslow M, Collins K, Robinson T, Green T, Madan J, et al. Health care professionals' preferences for extending mammographic breast screening to the over 70s. J Geriatr Oncol. 2011;2(1):1-10.

10. Parker L, Rychetnik L, Carter S. Values in breast cancer screening: an empirical study with Australian experts. BMJ open. 2015;5(5):e006333.

11. Parker LM, Rychetnik L, Carter SM. The role of communication in breast cancer screening: a qualitative study with Australian experts. BMC Cancer. 2015;15:741.
