# Supplementary material for: Do health professionals know about overdiagnosis in screening, and how are they dealing with it? A mixed-methods systematic scoping review
Source: PLoS One. 2025 Feb 3;20(2):e0315247. doi: 10.1371/journal.pone.0315247 (PMC11790174; doi:10.1371/journal.pone.0315247)
Supplement: S5 Table — GRADE CERQual. (DOCX) [file pone.0315247.s006.docx]

# GRADE-CERQUAL – assessment of confidence in the evidence

| **Summary of review findings** | **Studies contributing to the review finding** | **Methodological limitations** | **Coherence**  How clear and cogent is the fit between the data from the primary studies and a review finding that synthesises that data | **Adequacy**  We are likely to have concerns about data adequacy when we have concerns about the richness or the quantity of the data in relation to the claims made in the review finding. | **Relevance**  the extent to which the body of data from the primary studies supporting a review finding is applicable to the context specified in the review question | **CERQual assessment of confidence in the evidence** | **Explanation of CERQual assessment** |
| --- | --- | --- | --- | --- | --- | --- | --- |
| **Awareness & knowledge.**  Some HCPs seem to have a good understanding of what overdiagnosis is, while others seem to confuse it with false positives or other screening phenomena. | Clements[5]*  Dois[3]  Gimenez[1]  Parker[6, 10, 11]*  Pickles[7]*  Smith[4]  Toledo-Chávarri[8]*  Walters[9]* | ***Minor concerns***  The studies that attributed most to the finding are good quality studies. The other studies have some methodological issues due to unclarity of the basis for evaluative appraisal, underlying assumptions and theoretical perspectives. Some studies also have a very selected (biased) sample of participants. | ***Moderate concerns***  All relevant data support the review finding. However, the information is circumstantial because none of the studies intended to investigate this review question. | ***Moderate to major concerns***  Especially about the second component of this finding that some HCPs seem to confuse overdiagnosis with other concepts. Only 2 studies contributed to this component.  No concerns about the first part. | ***Moderate to major concerns*** Few studies with rich data. Only info on breast cancer (public health perspective) and prostate cancer (GP perspective). No info about specialist doctors. Possible selection bias (participants with higher than average level of interest in the topic). | **Low to moderate confidence** | All studies contribute some data to this finding, however few studies contribute rich data. All information is circumstantial. |
| **Perception of overdiagnosis**  HCPs vary substantially in the extent to which they consider overdiagnosis an important problem. The differences depend mainly on how HCPs perceive the potentially harmful consequences of overdiagnosis. Their perceptions of overdiagnosis seem intertwined with their beliefs about screening benefits and whether screening should be offered to the public. | Parker[6, 10, 11]*  Pickles[7]*  Toledo-Chávarri[8]  Walters[9] | ***No or very minor concerns*** The studies that attributed to this finding are good quality studies. | ***No or very minor concerns***  All relevant data support the review finding. | ***Moderate concerns***  Two good quality studies with rich data constitute the primary source of evidence supporting this finding. The 2 other studies provide (partial) confirmation. Overall, relatively few data. | ***Moderate concerns*** Only two studies with rich data (from the same research group, same country). The 2 other studies only provide indirect information. Only info on breast cancer (public health perspective) and prostate cancer (GP perspective). No info about specialist doctors. Possible selection bias (participants with higher than average level of interest in the topic). | **Low to moderate confidence** | Only 4 studies provide data for this finding, however 2 of which are high-quality studies with rich data. Uncertain to what extent this finding can be generalised to all HCPs and different forms of screening. |
| **Impact on screening policies** Depending on their perception of the harmful consequences of overdiagnosis and the degree to which they value the benefits of screening, HCPs tend to adhere to one of these three screening policies: avoiding screening, providing adapted screening and support informed decision making, and offering screening and encouraging people to participate in this screening. | Gimenez[1]  Parker[6, 10, 11]*  Pickles[7]*  Toledo-Chávarri[8] | ***No or very minor concerns*** The 3 studies that contributed most to this finding are good quality studies. | ***No or very minor concerns***  All relevant data support the review finding. | ***Moderate concerns***  Two good quality studies with rich data constitute the primary source of evidence supporting this finding. The 2 other studies provide (partial) confirmation. Overall, relatively few data. | ***Moderate concerns*** Only two studies with rich data (from the same research group, same country). 1 other study, also good quality provides some indirect information. Only info on breast cancer (public health perspective) and prostate cancer (GP perspective). No info about specialist doctors. Possible selection bias (participants with higher than average level of interest in the topic). | **Low to moderate confidence** | Only 4 studies provide data for this finding, however 2 of which are high-quality studies with rich data. Uncertain to what extent this finding can be generalised to all HCPs and to different forms of screening. |
| **HCPs' preparedness to provide information about overdiagnosis**  HCPs’ preferences for informing their patients or the public about overdiagnosis align with how they generally perceive the net benefit of the proposed screening, their attitude towards the phenomenon of overdiagnosis, and what "taking good care for patients or the public" means to them as professionals. How they communicate about this varies widely, with significant differences in terms of content, amount of detail and connotation of harm. | Clements[5]  Dois[3]  Gimenez[1]  Parker[6, 10, 11]*  Pickles[7]*  Smith[4]  Toledo-Chávarri[8]* | ***Minor concerns*** Four studies of good quality (three of them attributed most to this finding). The other studies have some methodological issues due to unclarity of the basis for evaluative appraisal, underlying assumptions and theoretical perspectives. Some studies also have a very selected (biased) sample of participants. | ***No or very minor concerns***  All relevant data support the review finding. | ***Moderate concerns***  Three good quality studies with rich data constitute the primary source of evidence supporting this finding. The other studies provide (partial) confirmation. Overall, relatively few data. | ***Moderate concerns*** Only three studies with rich data. Only info on breast cancer (public health perspective) and prostate cancer (GP perspective). No info about specialist doctors. Possible selection bias (participants with higher than average level of interest in the topic). | **Low to moderate confidence** | Seven studies provide data for this finding, 4 of them are high-quality studies, and 3 provide rich data for this finding. Uncertain to what extent this finding can be generalised to all HCPs and to different forms of screening. |

1. Gimenez L, Janczewski A. Representation of overdiagnosis in breast cancer screening among general practitioners: a qualitative study by focus groups. Exercer-La Revue Francophone De Medecine Generale. 2018(139):18-9.

2. Malli G. [Early detection of prostate cancer by PSA testing: the results of a qualitative study on barriers caused by physicians in Austria implementing informed decision making]. Gesundheitswesen (Bundesverband der Arzte des Offentlichen Gesundheitsdienstes (Germany)). 2013;75(1):22-8.

3. Dois A, Bravo P, Fernández-González L, Uribe C. [Experts' views on the communication of risks and benefits of mammography to detect breast cancer]. Rev Med Chil. 2021;149(2):196-202.

4. Smith J, Dodd RH, Wallis KA, Naganathan V, Cvejic E, Jansen J, et al. General practitioners' views and experiences of communicating with older people about cancer screening: a qualitative study. Fam Pract. 2022.

5. Clements A, Watson E, Rai T, Bukach C, Shine B, Austoker J. The PSA testing dilemma: GPs' reports of consultations with asymptomatic men: a qualitative study. BMC Fam Pract. 2007;8:1-7.

6. Parker LM, Rychetnik L, Carter S. Framing overdiagnosis in breast screening: a qualitative study with Australian experts. BMC Cancer. 2015;15:606.

7. Pickles K, Carter SM, Rychetnik L. Doctors' approaches to PSA testing and overdiagnosis in primary healthcare: a qualitative study. BMJ open. 2015;5(3):e006367.

8. Toledo-Chávarri A, Rué M, Codern-Bové N, Carles-Lavila M, Perestelo-Pérez L, Pérez-Lacasta MJ, et al. A qualitative study on a decision aid for breast cancer screening: Views from women and health professionals. European Journal of Cancer Care. 2017;26(3).

9. Walters SJ, Winslow M, Collins K, Robinson T, Green T, Madan J, et al. Health care professionals' preferences for extending mammographic breast screening to the over 70s. J Geriatr Oncol. 2011;2(1):1-10.

10. Parker L, Rychetnik L, Carter S. Values in breast cancer screening: an empirical study with Australian experts. BMJ open. 2015;5(5):e006333.

11. Parker LM, Rychetnik L, Carter SM. The role of communication in breast cancer screening: a qualitative study with Australian experts. BMC Cancer. 2015;15:741.
