# Supplementary material for: Do health professionals know about overdiagnosis in screening, and how are they dealing with it? A mixed-methods systematic scoping review
Source: PLoS One. 2025 Feb 3;20(2):e0315247. doi: 10.1371/journal.pone.0315247 (PMC11790174; doi:10.1371/journal.pone.0315247)
Supplement: Attachment — Submitted filename: Response to reviewers.docx [file pone.0315247.s007.docx]

Dear editor,

We thank the reviewer for their thoughtful reading, encouraging words and helpful feedback. We address all the points below (in italic)

1. Fundamental concepts and scope

“There appears to be a disjunct between the introduction and the rest of the manuscript; there are also some conceptual inconsistencies within the introduction.

**# 1 #**
First, the introduction presents one conception of overdiagnosis when there are a range of definitions or conceptualisations which have continued to evolve in recent years. Given that this paper is fundamentally about what HPs take overdiagnosis to be, suggest it is wise to start by recognising that the concept and its definition is contested even among experts (whether or not all experts recognise this contestation!).”

- *The debate about the concept, the semantics, the scope, and the solutions for this contentious phenomenon is indeed still ongoing. Although this systematic review (SR) is deliberately limited to overdiagnosis in the context of screening, it also occurs in a clinical setting, and even when confined to the setting of screening, experts may differ in how they would define overdiagnosis
  We added this notion to the introduction section.

  Changed text in MS:
  Line 75: “*Screening for disease or risk factors is the most important driver of overdiagnosis. Still, overdiagnosis might also occur in clinical practice through incidental findings, broadening diagnostic criteria, or overselling common life experiences as diseases or medical entities. The ever-growing emphasis on early diagnosis and fast-track diagnostic packages in cancer diagnosis steadily increases the risk of overdiagnosis in clinical care[12]. The concept is also often confused with other forms of medical overuse (overtesting, overtreatment) or with diagnostic errors (false positives, misdiagnosis)[1]. The debate around the definition and the conceptual challenges of overdiagnosis is still ongoing and elucidates different opinions about the semantics of the definition and the operationalisation of the concept. Some authors believe that a key feature of overdiagnosis is that the condition would never have led to symptoms if left undetected, while others state that also diagnoses that ultimately have no utility or result in more harm than benefit are overdiagnosed. Other points of debate are: should the notion of a potential benefit of overdiagnosis be allowed, and who should decide how harmful overdiagnosis is (the public or experts) [13-19]?”

Further, the introduction states that overdiagnosed people “cannot benefit from this early diagnosis” (p.3 L57) and that treating a person with an overdiagnosed condition “cannot provide any benefit” p. 3 L65), but also states that the harms caused by overdiagnosis “must be weighed against the benefits of screening” (p.4 L82). The manuscript itself presents nuanced accounts of the complex benefit/harm balance involved in understanding and identifying overdiagnosis. Arguably this nuance is central to the main summary figure, Fig2. I would suggest this more nuanced approach is more consistent with the reality of what clinicians (and their patients) face and should be reflected in the introduction.

- *We added some lines to the introduction to emphasise the difficult trade-off and uncertainty screening candidates and their HPs have to deal with when deciding about screening. We acknowledge that we emphasise the harms related to overdiagnosis. This is intentional, because this SR is not about HPs’ overall ideas about benefits and harms of screening, but deliberately zooms in on the (in our opinion) under-exposed problem of overdiagnosis.*

*Changed text in manuscript (line 95):*
Nevertheless, overdiagnosis is nowadays seen as a substantial harm of screening, which must be weighed against the potential benefits of screening, such as lower disease-specific mortality rates [24-26], reduced morbidity [27, 28], or preservation of functionality [29]. Screening is inevitably taking a change for the future: the decision to screen for a particular condition is made in the hope of benefiting from earlier diagnosis and treatment while avoiding possible negative consequences. At the time of screening, however, there is only uncertainty: about the individual risk of developing a detrimental disease, the chances of benefiting from an earlier diagnosis, and the risks of getting harmed due to screening. It is a difficult trade-off for individuals and the health professionals who guide them in these screening decisions. Eligible screening participants should be informed comprehensively and understandably about the benefits and harms of screening based on the best available evidence and encouraged to make an informed decision that aligns well with their preferences and values in health [30-34].

Finally, the manuscript uses the word screening without explaining how the study is conceptualising screening, or the imagined relationship between population screening and opportunistic testing. Your primary data seems to contain the views of both clinicians (who may be offering opportunistic tests to individuals, or may be informing individuals about population based screening programs) and administrators and others with an interest in population based programs. Suggest this screening-opportunistic testing distinction should be addressed directly, and you should make sure that when you report findings you are clear whether you are talking about a clinician (e.g. a GP) or an administrator (e.g. a program manager). This is especially pertinent to your third RQ which refers to ‘offering’ screening, but it is not completely clear what constitutes an offer in the context of the review and these different contexts.

- *We thank the reviewer for drawing our attention to the fact that we did not convey this clearly in our SR. Indeed, we intend to encompass all forms of screening, because overdiagnosis is an issue in both population-based screening and opportunistic screening. We changed our manuscript at several locations to make this more clear (see below), and we also added a column in table 1 (overview of the included publications) to indicate whether the study took place in a setting with an ongoing population-based screening program or not.*

*Changed text in manuscript*

*Methods-section (Line 145)*
We broadly defined HPs as all professionals who, in the course of their professional activities, are involved in organising, offering, or performing screening. This encompasses public health (PH) officials and experts involved in population-based screening programs and individual healthcare providers (IHPs), e.g. clinicians who may talk about or offer screening to individual patients. Likewise, the concept of screening is also broadly defined as all forms of testing among asymptomatic people to find a disease early to improve the prognosis by early treatment.

Description of the included publications (Line 242)

We found no studies on other forms of population-based screening (e.g. neonatal screening) nor on other forms of opportunistic screening that individual healthcare providers can offer in their consultations (e.g. screening for diabetes, STD, melanoma, etc. ).

All the studies about prostate cancer screening questioned HPs in their role as individual healthcare providers and inquired about their intentions and actions regarding offering opportunistic prostate cancer screening to their patients. These studies all took place in settings without population-based prostate screening programs and with different, sometimes contradicting, screening guidelines or professional recommendations. [60, 63, 67, 68, 71-74, 76-78]. The studies about the other types of screening (breast, colorectal, and cervical) were held in settings where population-based screening programs were in place. Some studies examined IHPs in their role as implementers of these screening programs, while others took a public health perspective, asking professionals about their ideas on organising mass screening and how to inform the public about it [61, 64-66, 69, 70, 75].

Results – RQ3

Depending on the HP’s role, these screening approaches may be differently operationalised. HPs who speak from a public health perspective express their ideas about how population-based screening programs should be organised, while IHPs explain how they act personally in interaction with their patients. Table 4 presents some citations illustrating the three screening approaches for the PH and the IHP perspective.

Note that addressing these comments is not just about editing the introduction, but also about reflecting on how the concepts were operationalised in the the course of undertaking the review, and being sure that these were internally consistent and are consistent with the finished Introduction.

2. Methods

As noted, the review is generally very strong.
There are reasons given for abandoning CIS in the discussion, but I wonder whether this information may be better in the methods? As there appears to be a significant amount of inductive analysis involved in the review, I was not 100% clear why CIS was abandoned. I was also not clear whether you would describe the methodology you followed as bespoke, or whether you found an alternate methodology? Some justification for why you did things the way you did them, if it was a bespoke method, would be helpful.

- *Thank you for this remark. We moved our explanation from the discussion to the methods section. After abandoning CIS, we did not literally follow a specific method . However, what we did aligns most with the method of Thematic Synthesis. We changed our methods section accordingly to convey our response to this remark.*

*Line 153*
In our protocol [53], we planned to analyse the findings with Critical Interpretive Synthesis (CIS) as the review method [57, 58]. However, it was not possible to apply this approach for several reasons. First, our research question was already explicitly defined from the beginning, which left little room for the inductive development of the research question and related iterative literature search, as promoted by CIS. Second, we found only a few studies that provide rich data, most studies mention overdiagnosis only marginally, which leaves too scarce material for generating new overarching concepts. Third, the data from the quantitative studies were too limited, fragmented, and heterogeneous to integrate them in a meaningful synthesis with the qualitative data. We, therefore, analysed the qualitative and quantitative data separately and present them in two complementary syntheses [59]. The analysis of the qualitative data was inspired by the principles of Thematic Synthesis [60], but we also kept some features specific to CIS, such as allowing several study designs, combining qualitative and quantitative data, and using the perspective of a multidisciplinary research team during data analysis.

Can you say more about why you didn’t assess quality of the quantitative studies?

- *Thank you for drawing our attention that we did not make it sufficiently clear that we did assess the quality of the quantitative studies (see table 2). However we did not assess the certainty of evidence we obtained from these questionnaire studies because we could not find any formal grading tool to assess the certainty of evidence from questionnaire studies*.

3. Findings

The knowledge/awareness results seemed thin, although this is arguably compensated by later sections.

The most substantive section is the ‘perception of overdiagnosis’ section. This section appears to rely heavily on a small number of the studies found based on the quotations used. If this is the case, suggest addressing this more directly.

- *We added to each section the number of studies our findings are based on.*

The important ‘perceptions of overdiagnosis’ section also appears to rely heavily on quotes rather than presenting very much new analysis from the authors of this review. Can you provide any further analytic depth that draws out new insights by comparing & further synthesising the primary studies?

*We believe our main analysis lies in the fact that HPs’ perception of overdiagnosis and how they deal with it cannot be separated from their beliefs in the benefits of screening. This analysis covers the entirety of the results. We moved this overarching analysis to the beginning of the results section in order to highlight the new in depth insights. .*

*Line 306*

## Overarching analysis: an inextricable interplay between the perception of overdiagnosis and overall beliefs about screening

The qualitative findings show that HPs familiar with the phenomenon of overdiagnosis vary substantially in the extent to which they consider it a significant problem, even when they use a similar definition for the concept. HPs’ perceptions of overdiagnosis (RQ2) appear inextricably intertwined with HPs’ broader beliefs about the benefits and harms of screening and whether screening should be offered to the public. HPs who pay much attention to the harmful consequences of overdiagnosis also tend to be more sceptical about the benefits of screening and are more reluctant to offer it. Conversely, HPs who strongly believe in the life-saving potential of screening and advocate that as many people as possible should be screened seem to see overdiagnosis as a minor issue with little or no health consequences. These beliefs are on a sliding scale, and the correlation between perceptions of overdiagnosis and general beliefs about screening cannot be considered a one-to-one match. These ideas also correlate with HPs’ position towards offering screening (see RQ3) and their willingness to discuss the risk of overdiagnosis (see RQ4). Figure 2 illustrates this overarching analysis in a schematic overview of how the perception of overdiagnosis is connected to overall screening beliefs, preferred screening policies, and willingness to communicate about overdiagnosis.

The quantitative and qualitative findings seem quite disconnected in the results section – is it possible to connect them any further?

*Thank you for this suggestion. Throughout the manuscript we tried to connect both types of results more on a content level, while still maintaining our segregated analysis, as described in the methods section.*

Figure 2 is useful and interesting and will be an important contribution of the study.

The discussion is a strength of the MS and raises some important and useful implications.

*The authors want to thank the reviewer for this positive comment.*
